# Degradation trends in lakes and wetlands of Iran and their contribution to dust pollution

**DOI:** 10.1038/s41598-026-40357-1

**Published:** 2026-02-18

**Authors:** Seyed Arman Samadi-Todar, Zohre Ebrahimi-Khusfi, Mohsen Ebrahimi-Khusfi

**Affiliations:** 1 Department of Remote Sensing, Faculty of Geographical Sciences, University of Tehran, Tehran, Iran; 2 Department of Environmental Science and Engineering, Faculty of Natural Resources, University of Jiroft, Jiroft, Iran; 3 Department of Geography, Yazd university, Yazd, Iran

**Keywords:** Wetland degradation, Dust aerosol, Environmental threats, Influencing factors, Remote sensing, Environmental sciences, Natural hazards

## Abstract

Analysis of change trends in lakes and wetlands (LWs) degradation, identification of the main driving forces, and exploration of their impact on dust pollution are of high importance. This research investigated these issues in 30 LWs in Iran. Landsat imagery sensors were used to calculate the wetland degradation index (WDI) during the period 1986–2024. Daily aerosol optical depth data were used to calculate dust event frequency (DEF). The trend of changes in the severely degraded bed (SDB) of LWs was analyzed using the Mann-Kendall test. The Mann-Whitney U test was used to compare the mean degraded area before and after the change point. The contribution of influencing factors to the SDB area was determined using Ridge Regression (RR), and their relationship with DEF was determined using Spearman’s correlation coefficient (*p*). The results showed that the area of SDB increased significantly in 23% of Iran’s LWs (Z > + 2.1; *p* < 0.05; t-test/Mann–Whitney U test). Changes in precipitation, wind speed, vapor pressure, surface pressure, albedo, land surface temperature, and runoff were the most important driving factors during the pre-change period. In the post-change period, the importance of these factors diminished across the majority of these critical wetlands, while the impact of population pressure around most wetlands increased. A weak positive correlation was observed between SDB and DEF in the Maharlu, Bakhtegan-Tashk, Meyghan, and Namak Lake (*p* <0.28; P-value > 0.05). However, a significant positive correlation was found between SDB and DEF in the three important wetlands of Gomishan (*p*= 0.55), Parishan (*p*= 0.35), and Namak Lake (*p*=0.70). A notable association was found between DEF variability and SDB dynamics, with considerable differences among wetlands. Effective management is necessary to protect and restore endangered wetlands and reduce dust hazards in Iran.

**Supplementary Information:**

The online version contains supplementary material available at 10.1038/s41598-026-40357-1.

## Introduction

Wetlands, one of the most important ecosystems on Earth, are crucial for climate, biodiversity, carbon sequestration, providing medicine and recreational opportunities^1–3^. In Iran, one of the countries located on the world’s dry belt, lakes and wetlands (LWs) play vital roles at national and regional levels. However, climate change and human activities have led to the degradation and reduction of wetland areas^4,5^, and ecological function have declined^4,6–8^. Considering that wetlands play irreplaceable roles on regional and global scales, their monitoring and investigation are essential for maintaining their ecological capacity and preserving these valuable ecosystems^9–11^.

Given the wide spatial distribution of wetlands and the temporal and spatial variations of their vegetation cover and water level, studying and investigating them using field methods is difficult, time-consuming, and costly^12–14^. Although remote sensing, and particularly the use of Landsat imagery, has become a useful and popular tool for the long-term monitoring of the ecological status of wetlands in various regions^15–17^, it is not without limitations. One of the limitations of Landsat imagery is its medium (30 m) spatial resolution, which may not be suitable for monitoring environmental changes in areas smaller than a single pixel^18–20^.However, since the studied wetlands have a surface area larger than 0.9 km², this limitation does not affect the present study. Moreover, cloud cover can influence the accuracy of surface reflectance^21,22^; to overcome this limitation, only images with less than 10% cloud coverage were used in this study. In addition, the Scan Line Corrector (SLC) error, which has occurred in Landsat 7 ETM+ images since 2003^23,24^, is another limitation that was addressed in this study using a Gap Filling correction method^24^.

In some studies, remote sensing-based ecological indices have been successfully used to monitor the status of wetlands in different regions of the world^25–27^. Recently, Zhu et al.^28^combined the lake shrinkage index (LSI), vegetation degradation index (VDI), and soil degradation index (SDI) extracted from the Landsat time-series images, and introduced the wetland degradation index (WDI) as an efficient index for assessing the severity of wetland degradation in Bashang Plateau. It has also proven useful for monitoring Alpine wetlands^29^. Generally, the WDI has shown promising results in different environmental regions, including alpine and plateau wetlands; however, its application in arid and semi-arid regions such as Iran remains limited. In particular, the spatial expansion of severely degraded beds (SDBs), as identified by the WDI, has not yet been assessed for Iranian lakes and wetlands.

Although the sensitivity of the WDI to seasonal variations, especially when images from different times of the year are used, is a limitation in the application of this ecological index, this constraint was overcome by utilizing images from dust-prone seasons. This ensured temporal consistency when assessing the impact of wetland degradation on dust events.

Multiple studies have been conducted on wetlands in Iran using remote sensing techniques. Surveys conducted on the trend of water level changes in Iranian wetlands during the 1990–2022 period indicate that, with the exception of Bakhtegan and Shurgol, which experienced an increase, all other wetlands faced a decrease^30^. The reduction in the water level of Gavkhouni, Parishan, and Jazmourian wetlands during the 1988–2018 period was reported by Ebrahimi-Khusfi et al.^31^. The decrease in the water level of Hamoun wetland during the 1990–2020 period was also confirmed by Zolfaghari et al.^8^. However, an increasing trend in water level has also been documented for the Hur al-Azim in spring and for the Shadegan wetland in winter^31^. These studies have primarily focused on changes in water levels and hydrological stresses rather than on the severity of degradation resulting from desiccation. This is an issue that receives particular attention in the present study.

Land use changes, dam constructions, and the unsustainable extraction of surface and groundwater resources in Iran, have played a significant role in the desiccation of LWs^32–35^. However, detailed information on these factors is not available for all the studied LWs in Iran. Considering that population is the primary factor influencing human activities around aquatic ecosystems in arid and semi-arid areas^36,37^, this study examined population trends as a proxy for the most significant human-based factor in different buffer zones.

One of the consequences of wetland degradation is the increased emission of dust from their dried beds, which affects the health of people and the surrounding environment^38–40^. Some studies have attributed the dust event frequency (DEF) to the reduction in the water surface area of nearby wetlands^41–44^. However, the impact of the severely degraded beds (SDB) of LWs, as derived from the WDI, on the DEF has not been investigated in Iran. Therefore, the present study seeks to address the identified research gaps. The main objectives are to (i) analyze the long-term change trend of the SDB in the national and international LWs in Iran; (ii) identify wetlands showing a significant increasing trend in the SDB area; (iii) identify the key factors affecting their degradation before and after the change point; (iv) investigate the impact of the SDB of critical wetlands on DEF.

## Study area

**Fig. 1:**
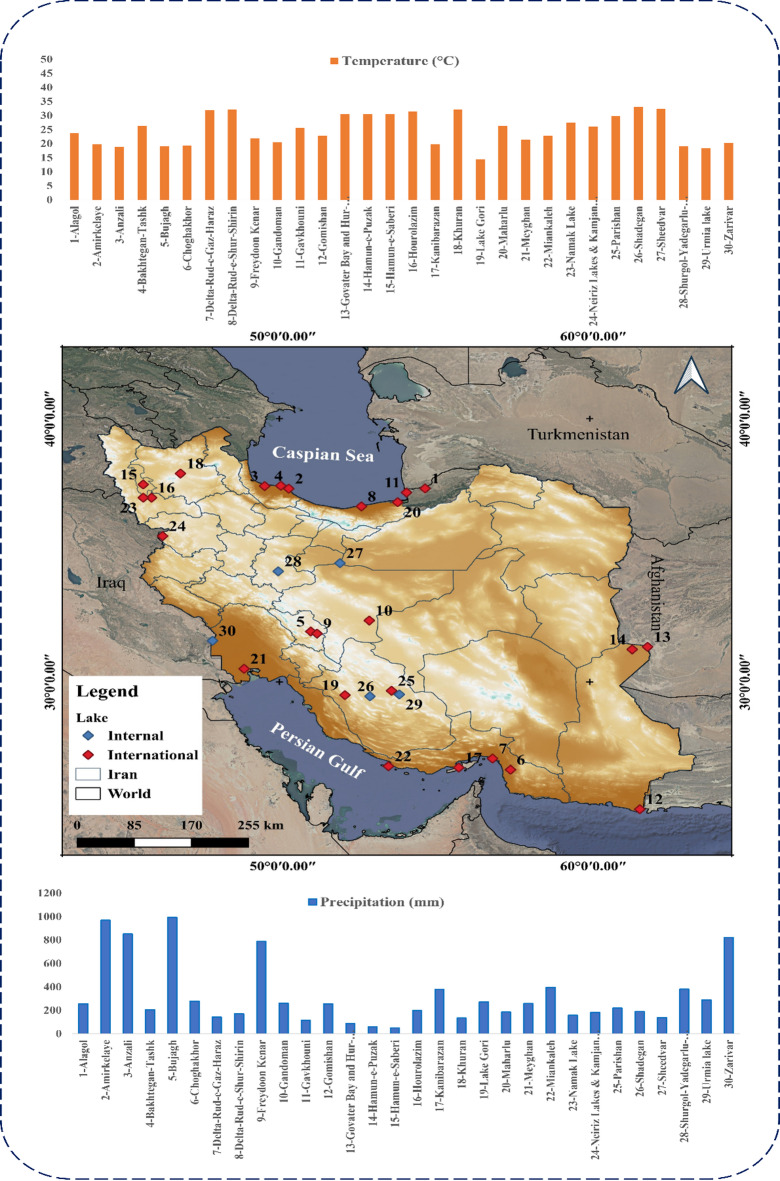
Spatial Distribution of Lakes and Wetlands and their long-term average temperature and precipitation (1986–2024) in Iran. Map created using the free and open-source software QGIS 3.40.12-Bratislava (QGIS Development Team; https://qgis.org/download/). The base map was obtained via the HCMGIS plugin (HCMGIS - Basemaps, Download Open Data, Batch Converter, VN-2000 Projections, and Field Calculation Utilities; https://plugins.qgis.org/plugins/HCMGIS/). Digital Elevation Model (DEM) data were sourced from SRTM 90 m (NASA Shuttle Radar Topography Mission; https://developers.google.com/earth-engine/datasets/catalog/CGIAR_SRTM90_V4). Digital Elevation Model (DEM) data were sourced from SRTM 90 m (NASA Shuttle Radar Topography Mission; https://developers.google.com/earth-engine/datasets/catalog/CGIAR_SRTM90_V4).

Iran is a country in the Middle East with an area of 1.648 million square kilometers, which has an arid and semi-arid climate^45^. Most of its land is located in the arid region of the world^7^. There are 84 important wetlands in Iran, covering a total area of over 20 million hectares^46^, of which 32%, with an area of about 1.5 million hectares, are registered in the Ramsar Convention (https://rsis.ramsar.org/. To investigate the objectives of the present study, 25 international and 5 important national lakes/wetlands in Iran were selected. The geographical distribution of the studied wetlands is shown in Fig. 1. The long-term (1986–2024) average rainfall has varied from approximately 60 mm in the Hamoun Wetlands located in southeastern Iran to over 850 mm in the Bujagh, Amirkelaye, and Anzali Wetlands in the north. According to the TerraClimate datasets, during the study period, the air temperature of the wetlands and lakes has ranged from about 14 °C in Lake Gori to over 33 °C in the Shadegan Wetland, in the southwestern of Iran. Based on the Shuttle Radar Topography Mission (SRTM) imagery, the elevation above sea level in the study areas ranges from − 31 m in Anzali Wetland to 2467.4 m above sea level in Gandoman Wetland.

## Data used and methods

**Fig. 2:**
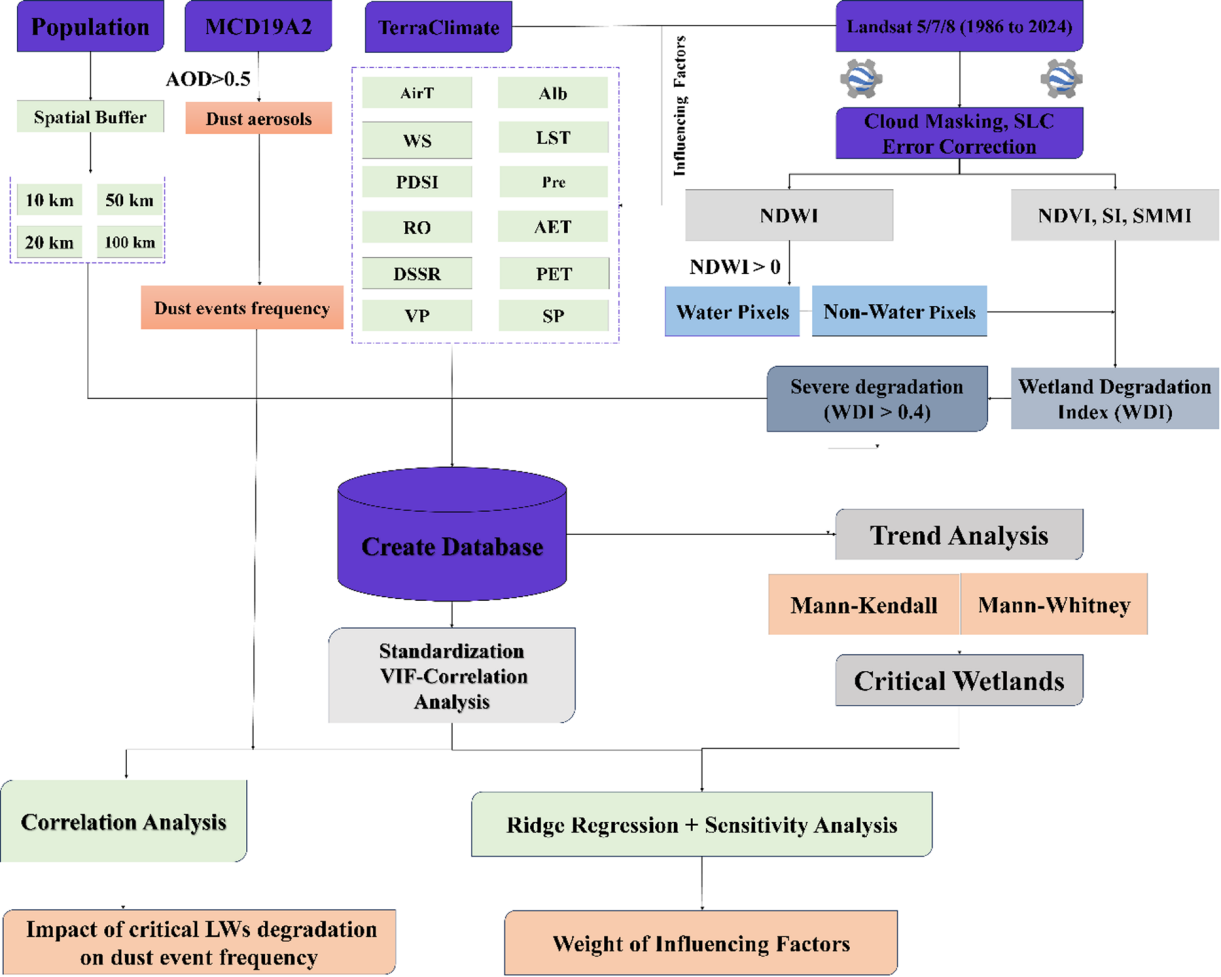
Research methodology flowchart for current study.

Figure 2 shows the current research methodology, and detailed descriptions of each method are presented below.

In this figure, AirT (Air Temperature), LST (Land Surface Temperature), Pre (Precipitation), WS (Wind Speed), AET (Actual Evapotranspiration), PET (Reference/Potential Evapotranspiration), RO (Runoff), DSSR (Downward Surface Shortwave Radiation), VP (Vapor Pressure), and SP (Surface Pressure), PDSI (Palmer Drought Severity Index), Alb (Albedo), the aerosol product MCD19A2 (MODIS Combined Daily Land Aerosol Optical Depth), NDVI (Normalized Difference Vegetation Index), NDWI (Normalized Difference Water Index), SMMI (Soil Moisture Monitoring Index), SI (Soil Salinity Index), SLC (Scan Line Corrector), LWs (Lake and Wetlands), WDI (Wetland Degradation Index), VIF (Variance Inflation Factor) are defined.

### Data used

The data used in the present research are divided into three categories, which include the following:

#### Landsat satellite surface reflectance imagery

Surface reflectance is a criterion of the proportion of solar radiation that reaches the Earth’s surface and returned to the Landsat sensor^47^. The LaSRC and LEDAPS surface reflectance algorithms correct the temporally, spatially, and spectrally varying scattering and absorbing influences of atmospheric gases, aerosols, and water vapor, which are necessary for making confident characterizations of the Earth’s land surface^48^.

Landsat satellite images have been available every 16 days since 1972. Considering that Landsat Collection 2 Level 2 images with high resolution and separability are available, they were selected for calculating the WDI (the calculation method of which is explained below). The required time series of Landsat 5, 7, and 8 satellite surface reflectance imagery for the 39-year period (1986–2024) were downloaded through coding on the Google Earth Engine platform (https://earthengine.google.com), the general specifications of which are mentioned in Table 1.

**Table 1:** Landsat satellite imagery utilized in this study.

Satellite / Sensor	Year	Collation / level	Pre‑Processing
Landsat- 5 (TM)	1986–2003	C2 / Level2	Surface Reflectance
Landsat- 5 (ETM+)	2003–2013	C2 / Level2	Surface Reflectance + SLC
Landsat- 5(OLI)	2013–2025	C2 / Level2	Surface Reflectance

**Fig. 3:**
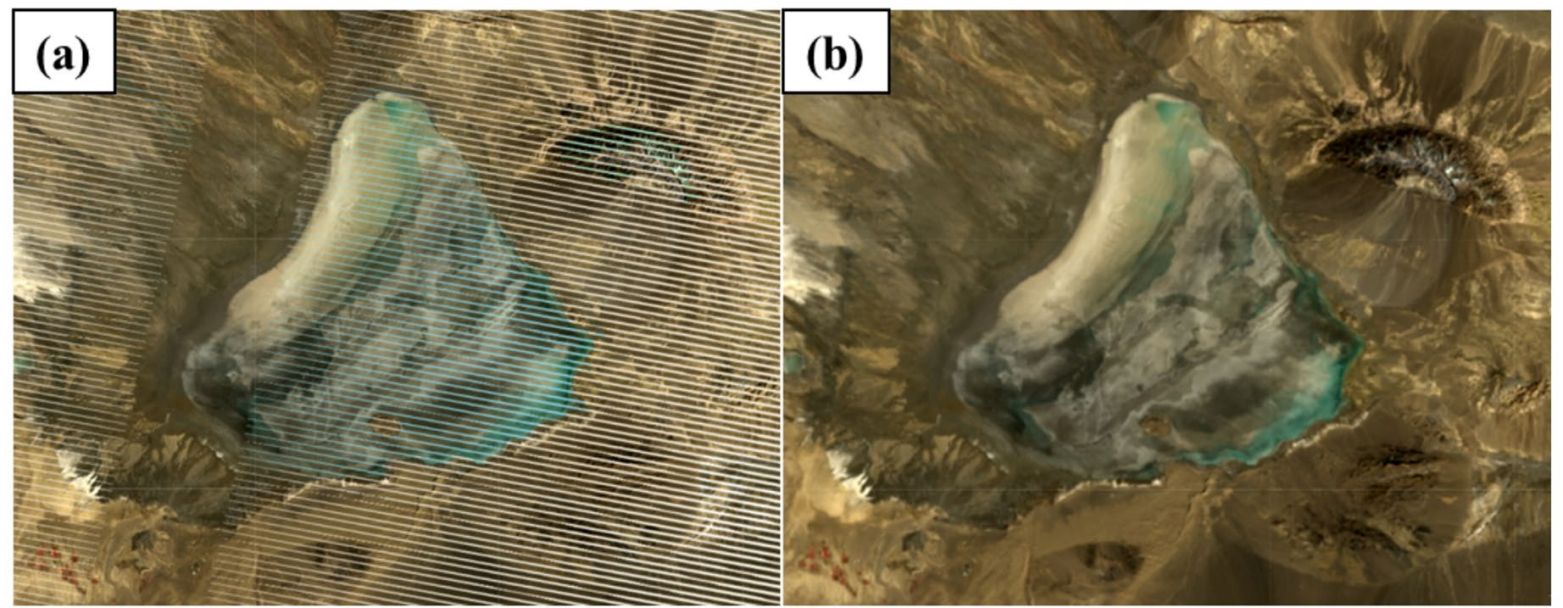
Lake Namak image before (**a**) and after (**b**) Scan Line Corrector error correction. Original satellite imagery was obtained from the USGS/NASA Landsat 7 program via Google Earth Engine (https://earthengine.google.com). Map created using the free and open-source software QGIS 3.40.12-Bratislava (QGIS Development Team; https://qgis.org/download/).

It is worth noting that the selected images were those with less than 10% cloud cover, and images with noisy errors such as shadow and cirrus clouds were masked using the Landsat quality band. Those Landsat 7 satellite images that had Scan Line Corrector (SLC) errors were also corrected on the Google Earth Engine platform. An example of this error correction over Lake Namak is shown in Fig. 3. Given the 30-meter spatial resolution and radiometric stability of Landsat surface reflectance products, these images are a suitable tool for monitoring and detecting changes in vegetation and water surface area at scales larger than 0.9 km^2^. These images have also been successfully used in previous studies^29,30,49–51^, which confirms their ability to capture changes in wetland ecosystems larger than the Landsat pixel size. Therefore, these images were used in the current study to calculate the WDI index and analyze the trend of changes in their dried beds.

#### Factors influencing LWs degradation

Considering the vital role of wetlands in maintaining microclimate balance in various regions, controlling floods, preserving biodiversity, stabilizing coastlines, and preventing erosion^52^, in addition to examining the status of wetlands, the key factors affecting their degradation should also be investigated. As mentioned in the introduction, numerous factors influence wetland degradation. Based on a comprehensive review of prior literature on wetland studies^53–55^, and also considering the availability of necessary data for the LWs in this study, factors such as precipitation, actual evapotranspiration, potential evapotranspiration, air temperature, drought index, runoff, downward surface shortwave radiation, vapor pressure, surface pressure, wind speed, albedo, and land surface temperature were selected. The specifications of the sensors and related products are presented in Table 2. Data for all environmental variables were extracted for the dusty seasons to maintain temporal consistency with the target variable. Also, winter averages of precipitation and runoff were calculated and used due to their importance in the hydrological system of the wetlands.

**Table 2:** Dataset specifications for factors influencing lake and wetlands condition.

Influencing factors	Sensor	Units	Spatial resolution	Temporal Resolution	References
Albedo (Alb)	Landsat 5/7/8	---	30 m	16 days	USGS
Land surface temperature (LST)	Landsat 5/7/8	°K			
Precipitation (Pre)	TerraClimate	mm	4638.3 m	Daily	^56^
Actual evapotranspiration (AET)	TerraClimate	mm			
Reference evapotranspiration (PET)	TerraClimate	mm			
Air temperature (AirT)	TerraClimate	°C			
Wind Speed (WS)	TerraClimate	m/s			
Palmer Drought Severity Index (PDSI)	TerraClimate	---			
Runoff (RO)	TerraClimate	mm			
Downward surface shortwave radiation (DSSR)	TerraClimate	W/m^2			
Vapor pressure (VP)	TerraClimate	kPa			
Surface Pressure (SP)	ERA5	Pa	27,830 m	Daily	^57^
Global population surface	JRC/GHSL		100 m	Annually	^58^

#### Moderate resolution imaging spectroradiometer imagery (MODIS) AOD product

The MCD19A2 product, provided by the Moderate Resolution Imaging Spectroradiometer (MODIS) at a daily temporal scale and with a spatial resolution of one kilometer, was used to extract dust aerosols (AOD > 0.5) over 30 studied LWs from 2000 to 2024 through the Google Earth Engine system (https://earthengine.google.com). This product, which extracts the concentration of atmospheric aerosols in two (blue and green) bands (0.47 and 0.55 μm) based on the Multi-Angle Implementation of Atmospheric Correction (MAIAC), was downloaded from 2000 to 2024 through the Google Earth Engine system and used to calculate the DFE as described below.

### Methodology

#### Water extent extraction and WDI calculation

In this study, the Normalized Difference Water Index (NDWI) was used to extract water bodies and pixels containing water, and to examine the inundation and drying of pixels^49^The NDWI index is one of the remote sensing indices in the field of identifying water bodies, which is calculated using Eq. (1), as follows^49,59^:

1 $$\:NDWI=\frac{({\rho\:}_{Green}-{\rho\:}_{NIR})}{({\rho\:}_{Green}+{\rho\:}_{NIR})}$$

Here, $\:{\rho\:}_{NIR}$ and $\:{\rho\:}_{Green}$ refer to the near infrared band and the green band.

**Fig. 4:**
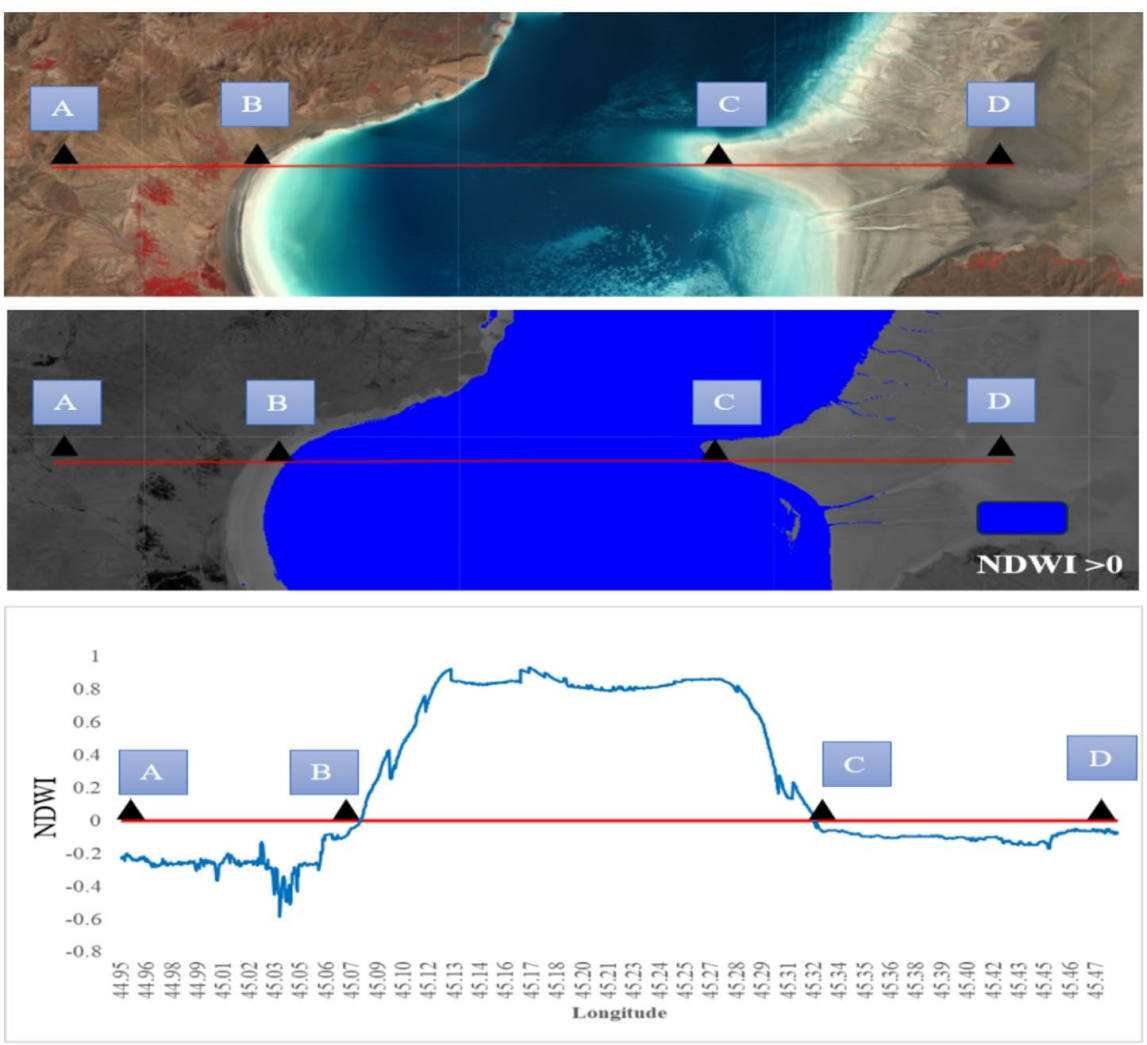
Changes in the NDWI along a cross-sectional transect of the wetland. (**a**) satellite image of the area, (**b**) water bodies identified with an NDWI value > 0, and (**c**): NDWI value changes along the longitude. points A, B, C, and D, are corresponding spatial locations in all three images. The base satellite imagery was obtained from the USGS/NASA Landsat 8 program via Google Earth Engine. Data processing and NDWI calculations were also performed in Google Earth Engine(https://earthengine.google.com). Map created using the free and open-source software QGIS 3.40.12-Bratislava (QGIS Development Team; https://qgis.org/download/).

Although the NDWI threshold value may vary depending on local conditions such as water turbidity, depth, and substrate characteristics, this study used a threshold of NDWI > 0, the effectiveness of which has been reported in previous studies^59,60^and confirmed by visual inspection of true-color images (Fig. 4).Finally, water pixels were considered as the number 1, and dry pixels as the number 0. This process was implemented on all annual Landsat images from 1986 to 2024 for 30 studied LWs. The water surface area extracted by the NDWI was considered as the non-degraded region of the lake and wetland.

The Wetland Degradation Index (WDI), proposed by Zhu et al.^28^, is calculated by combining three sub-indices: the Lake Shrinkage Index (LSI; Eq. 2), the Vegetation Degradation Index (VDI; Eq. 3), and the Soil Degradation Index (SDI; Eq. 4), as follows:

2 $$\:LSI=\frac{T}{N+1}$$

where T denotes the length of duration that the pixel was identified as non-water since it was detected as non-water for the first time, and N denotes the number of times that the pixel transitioned from non-water to water.

3 $$\:VDI=1-{NDVI}_{90p}$$

Here, NDVI90p denotes the maximum NDVI value at the 90th percentile.

In this study, the NDVI index was used to assess the vegetation cover status during the growing season (April 1st – September 30th).

4 $$\:\mathrm{S}\mathrm{D}\mathrm{I}=\left(0.5\times\:{\mathrm{S}\mathrm{M}\mathrm{M}\mathrm{I}}_{50\mathrm{p}}+0.5\times\:{\mathrm{S}\mathrm{I}}_{\mathrm{P}50\mathrm{p}}\right)$$

where $\:{\mathrm{S}\mathrm{M}\mathrm{M}\mathrm{I}}_{50p}$ and $\:{\mathrm{S}\mathrm{I}}_{50p}$ the normalized SMMI (Eq. 5) and SI (Eq. 6) at the 50th percentile, respectively.

5 $$\:SMMI=\frac{\sqrt{{{\rho\:}_{Red}}^{2\:}+\:{{\:\rho\:}_{NIR}}^{2}}}{\sqrt{2}}$$

6 $$\:SI=\sqrt{{\rho\:}_{Blue}\:\times\:\:{\rho\:}_{Red}}\:$$

Here, $\:{\rho\:}_{Red}$, $\:{\rho\:}_{NIR}$, and $\:{\rho\:}_{Blue}$ are the red, near infrared, and blue band, respectively.

Finally, WDI was calculated for all pixels using Eq. 7:

7 $$\:WDI=\left(0.6483\times\:LSI+0.2297\times\:VDI+0.1220\times\:SDI\right)$$

WDI values between 0 and 0.2, 0.2–0.4, 0.4–0.6, 0.6–0.8, and greater than 0.8 respectively indicate very slight, slight, moderate, severe, and very severe wetland degradation. In this study, pixels with WDI values greater than 0.4 were considered as the severely degraded bed (SDB) of the LWs. Finally, the trend in the changes of the SDB area was analyzed using the Mann-Kendall test as described in the next section.

It is worth noting that in this study, in order to reduce the sensitivity of the WDI to seasonal changes and maintain temporal consistency, satellite images from April to September, which coincide with the seasons prone to dust occurrence in Iran, were used. This approach also minimizes the effects of short-term seasonal fluctuations on the calculation of the LSI, VDI, and SDI sub-indices, especially changes caused by vegetation phenology and seasonal hydrological changes.

### Trend analysis

The Mann-Kendall test is one of the most widely used tests in the non-parametric method, presented by Mann^61^and developed by Kendall^62^. A significant advantage of this test is its ability to effectively detect the start of a trend, and it also has a wide range for identifying changes and can measure trends with high accuracy^63^. Therefore, it is suitable for data that do not follow a normal distribution, such as information related to weather and water resources^64^. In this paper, time series data (x1,x2,…,xn) are considered as random variables with a sample size of n, and the Mann-Kendall (M-K) test statistic in its standard distribution is defined as follows:

8 $$Z = \left\{ {\begin{array}{*{20}c} {\frac{{S - 1}}{{\sqrt {\mathrm{var} \left( S \right)} }},} & {S > 0} \\ {0,} & {S = 0} \\ {\frac{{S - 1}}{{\sqrt {\mathrm{var} \left( S \right)} }},} & {S < 0} \\ \end{array} } \right\}$$

9 $$\:S=\sum\:_{i=1}^{n-1}\sum\:_{k=i+1}^{n}sgn\left({X}_{k}-{X}_{i}\right)\:)$$

10 $$\mathrm{sgn} \left( {X_{k} - X_{i} } \right) = \left\{ {\begin{array}{*{20}c} {1,} & {X_{k} - X_{i} > 0} \\ {0,} & {X_{k} - X_{i} = 0} \\ { - 1,} & {X_{k} - X_{i} < 0} \\ \end{array} } \right\}$$

Where S is the Mann-Kendall test statistic, xi and xj are yearly values in years i and j (j > i), n is the length of data series, and sgn is sign (+ or −) of (xj - xi). Also, Z is the standard test statistic. Z statistic positive values show increasing trend and Z negative values show a decreasing trend in the time series. The null hypothesis of no trend is rejected if |Z| > Zα., where |Z| and α are the absolute value of the Mann-Kendall statistic and the level of statistical significance, respectively.

In the next step, the Pettitt test^65^was used to identify the change point, which allowed for the determination of the mean degraded wetland area before and after the change point. Subsequently, for the wetlands whose time-series data were normal, the t-test^66^was used, and for those that were non-normal, the Mann-Whitney U test^67^was employed to compare the means before and after the change point. The goal was to evaluate the accuracy of the results obtained from the Mann-Kendall test.

Finally, the factors influencing the changes in the SDB of the wetlands during the two periods (before and after the change point) were identified and analyzed using Ridge Regression (RR) analysis.

### Identifying key factors affecting the intensification of LWs degradation

Ridge regression (RR) is one of the methods for determining the importance of variables affecting the target variable, where factors with the greatest impact on the target function receive higher weights^68^. As the number of independent variables increases, the problems of multicollinearity and overfitting emerge, and reducing the number of variables will increase the variance of the final model^69^. Methods to overcome these issues include using Variance Inflation Factor (VIF) analysis and RR^70^. Since in this method, the coefficients of the regression model are determined based on the amount of penalty, the data must be standardized to ensure fair penalties. Therefore, in this research, the data were first standardized according to Eq. (11), and then, to minimize the impact of multicollinearity, VIF analysis (Eq. 12) was performed.

11 $$\:{Z}_{i}=\frac{{X}_{i-}\mu\:}{\sigma\:}$$

In the formula above, Z_i_ is the standard score for data X_i_, µ is the mean and σ is the standard deviation of the data. By doing this, the Z_i_’s will have a mean of 0 and a variance of 1.

12 $$\:{VIF}_{i}=\:{(1-{R}_{i}^{2})}^{-1}$$

In which, $\:{R}_{i}^{2}\:$is the determination coefficient obtained by regressing the ith predictor on the other predictor variables.

VIF values higher 4 indicate a high correlation between two independent variables, and such variables should be discarded to avoid reducing modeling accuracy.

Finally, ridge regression (RR) was used to determine the importance of the variables affecting the lake-wetlands degradation due to the short length of the statistical periods corresponding to the time intervals before and after the change point. The RR estimator is indicated by the k parameter (biasing parameter) that varies between 0 and 1. Using RMSE and AIC are among the common methods for determining the value of the ridge parameter^71^. The minimum value of these criteria indicates a better fit of the patterns and is calculated as follows^72^:

13 $$\:{{\upbeta\:}}^{\mathrm{*}}\left(\mathrm{k}\right)=({\mathrm{X}}^{{\prime\:}}\mathrm{X}+\mathrm{k}\mathrm{I}{)}^{-1}{\mathrm{X}}^{{\prime\:}}\mathrm{Y}$$

In this equation, β*(k) and k are the vector of standardized RR weights and biasing parameter, respectively. Also, I parameter refers to the p-dimensional identity matrix and *X*′*Y* denotes the predictor criterion correlation vector.

Since the ridge model primarily examines linear relationships between independent and dependent variables, sensitivity analysis was also employed to assess the potential presence of non-linear relationships and complex interactions among the variables. For this purpose, the Morris Method was employed in this research, which is a suitable approach for assessing the influence of input variables on the model’s output^73^. In this method, a set of samples is first generated from the input space based on the variation range of each variable. Then, the trained model is run for each combination of inputs, and the changes in the output relative to the step-by-step changes in each input are calculated. The overall importance of each variable is determined based on the mean of the absolute values of the elementary effects (µ*), and its degree of non-linearity or its interactions with other variables is determined using the standard deviation of these effects (σ). In this method, high values for both statistics indicate the presence of non-linear relationships or interaction effects among the variables^73,74^.

To investigate the impact of population dynamics as one of the most significant factors influencing changes in human activities^36,37^, we examined the five-year trends in the population of residents around critical wetlands within buffer zones of 10 to 100 km. For this purpose, global population surface imagery from 1985 to 2024 was used. In this study, the buffer zone is defined as the geographical area surrounding each water body, delineated by specific radii from its boundaries, and used specifically to assess the impact of population factors on the trend of degraded bed changes.

The buffer zones had a radius that ranged between 10 km and 100 km so that the extent to which the pressure exerted by human population size during the research period was mainly around the degraded wetlands, rather than distributed across the wider regions, could be investigated. Since the data concerning the global human population is provided every five years, correlation analysis between population growth and changes in the area of degraded wetlands was also considered within corresponding time intervals. Accordingly, following an assessment of data normality, Pearson’s correlation coefficient^75^ (Eq. 14) was applied to variables with normal distributions, while Spearman’s rank correlation coefficient^76^ (Eq. 15) was used for variables with non-normal distributions.

14 $$\:\mathrm{r}=\frac{{\sum\:}_{i}^{n}({X}_{i}-\overline{X})({Y}_{i}-\overline{Y})}{\sqrt{{\sum\:}_{i}^{n}({X}_{i}-\overline{X}{)}^{2}{\sum\:}_{i=1}^{n}({Y}_{i}}-\overline{Y}{)}^{2}}$$

15 $$\:\rho\:=1-\frac{6\sum\:{d}_{i}^{2}}{n({n}^{2}-1)}$$

Here, r and $\:\rho\:$ are the Pearson and Spearman’s rank correlation coefficient, respectively. In this study Xi and Yi are SDB and population growth rate, respectively. $\bar {X}$ represents the long-term average of the SDB area, $\bar {Y}$ represents the average of population growth rate in the time period from 1985 to 2024, and n is the number of years in the study period. Also, di is the difference between the ranks of the i^th^ pair of observations.

### Analysis of the impact of critical LWs degradation on dust event frequency

#### Dust events frequency

In current research, daily MODIS-AOD products at 550 nm wavelength were downloaded from 2000 to 2024. For every date, a binary image was created where pixels with AOD > 0.5, which represent dust aerosols, were given a value of 1, and all other pixels were given a value of 0^77,78^. Then, the frequency of dust events (events with AOD > 0.5) was calculated separately for each year across SDB of the critical LWs as follows^79^ (Eq. 16):

16 $$\:DEF=\frac{\sum\:_{1}^{365}(nAOD>0.5)}{\sum\:_{1}^{n}N}\:\times\:100$$

In this equation, DEF refers to the percentage of dust event frequency for a given year in the SDB. nAOD > 0.5 represents the total number of events with an aerosol optical depth value greater than 0.5, and N denotes the total number of observations within the SDB.

#### Correlation analysis between DEF and degraded LWs area (DLWA)

The correlation coefficient is a statistical index used to analyze the strength and direction of the linear relationship between two quantitative variables^80^. The most common type of correlation coefficient used to measure the linear relationship between two quantitative variables with an approximately normal distribution is the Pearson correlation coefficient (Eq. 15)^75^, and for non-normal distributions, it is the Spearman correlation coefficient (Eq. 16)^76^. At this stage of the study, these coefficients were used to analyze the relationship between SDB and DEF in Iran’s critical wetlands.

The applied software used in this study is presented in the Supplementary Material (Table S1).

## Results

### Spatiotemporal changes in severely degraded areas of LWs

**Fig. 5:**
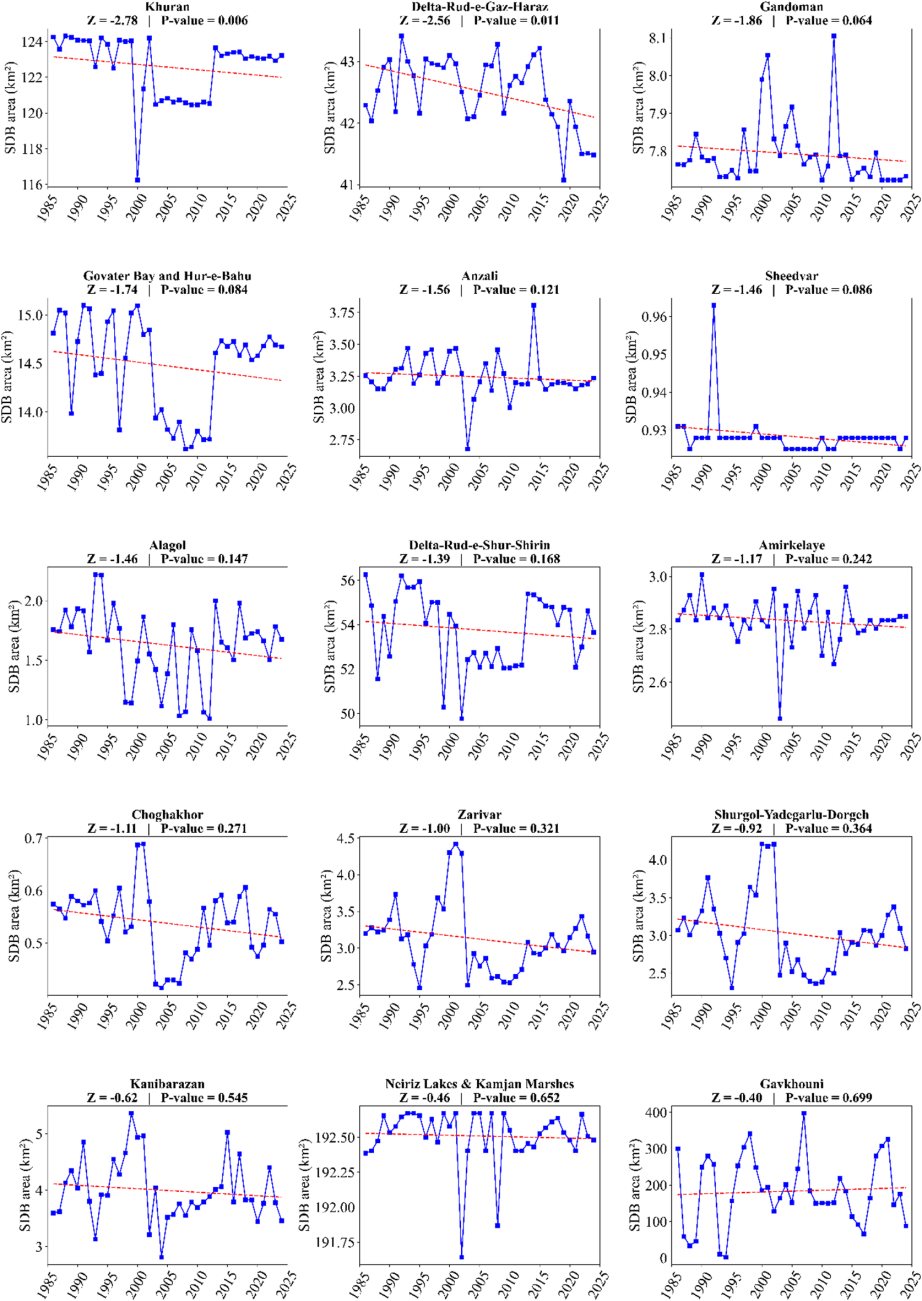
Annual changes in the Severely Degraded Bed (SDB) area of Iran’s wetlands and lakes during dusty periods (April–September) for the period 1986–2024. Z and P-value denote the Mann-Kendall statistic and significance level, respectively.

Using the Mann-Kendall test, we investigated changes in the SDB area of LWs in Iran. The results indicated that the destruction level of ten of the the SDB area showed a significant increasing trend. Conversely, two of them, namely Delta-Rud-e-Gaz-Haraz (Z = -2.6) and Khuran (Z = -2.8), showed a significant decreasing trend during the period 1986 to 2024 (Fig. 5). Specifically, Gomishan (Z = 4.6), Parishan (Z = 5.1), Shadegan (Z = 2.6), Urmia Lake (Z = 5.7), Maharlu (Z = 2.4), Namak Lake (Z = 2.1), Meyghan (Z = 2.6), Bakhtegan-Tashk (Z = 3.8), Hamun-e-Puzak (Z = 2.1), and Hamun-e-Saberi (Z = 2.4) all experienced a significant increasing trend during this period (Fig. 5).

**Fig. 6:**
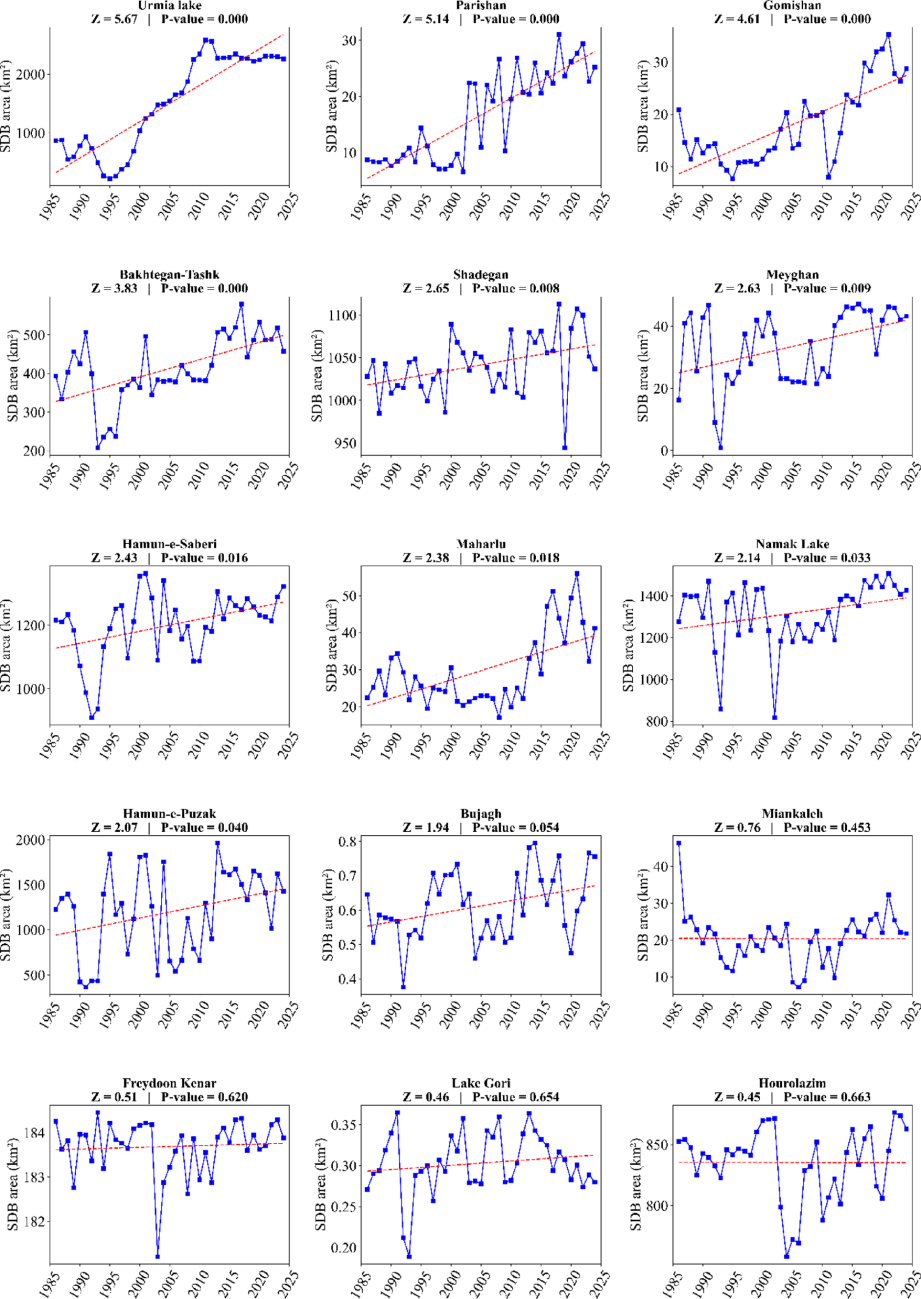

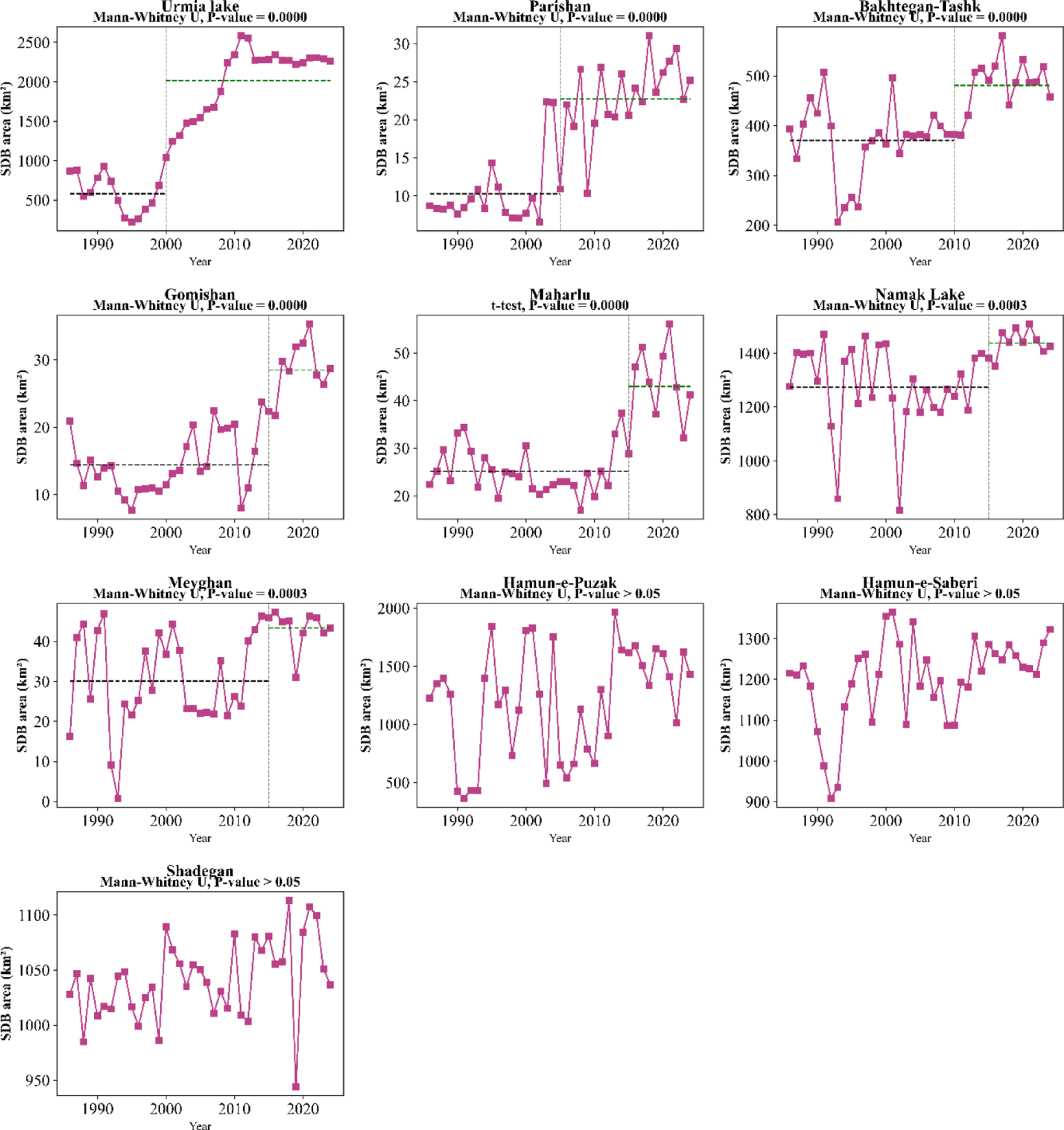
Change points in the severely degraded bed (SDB) area of the identified critical lakes and wetlands during the period 1986–2024.

**Fig. 7:**
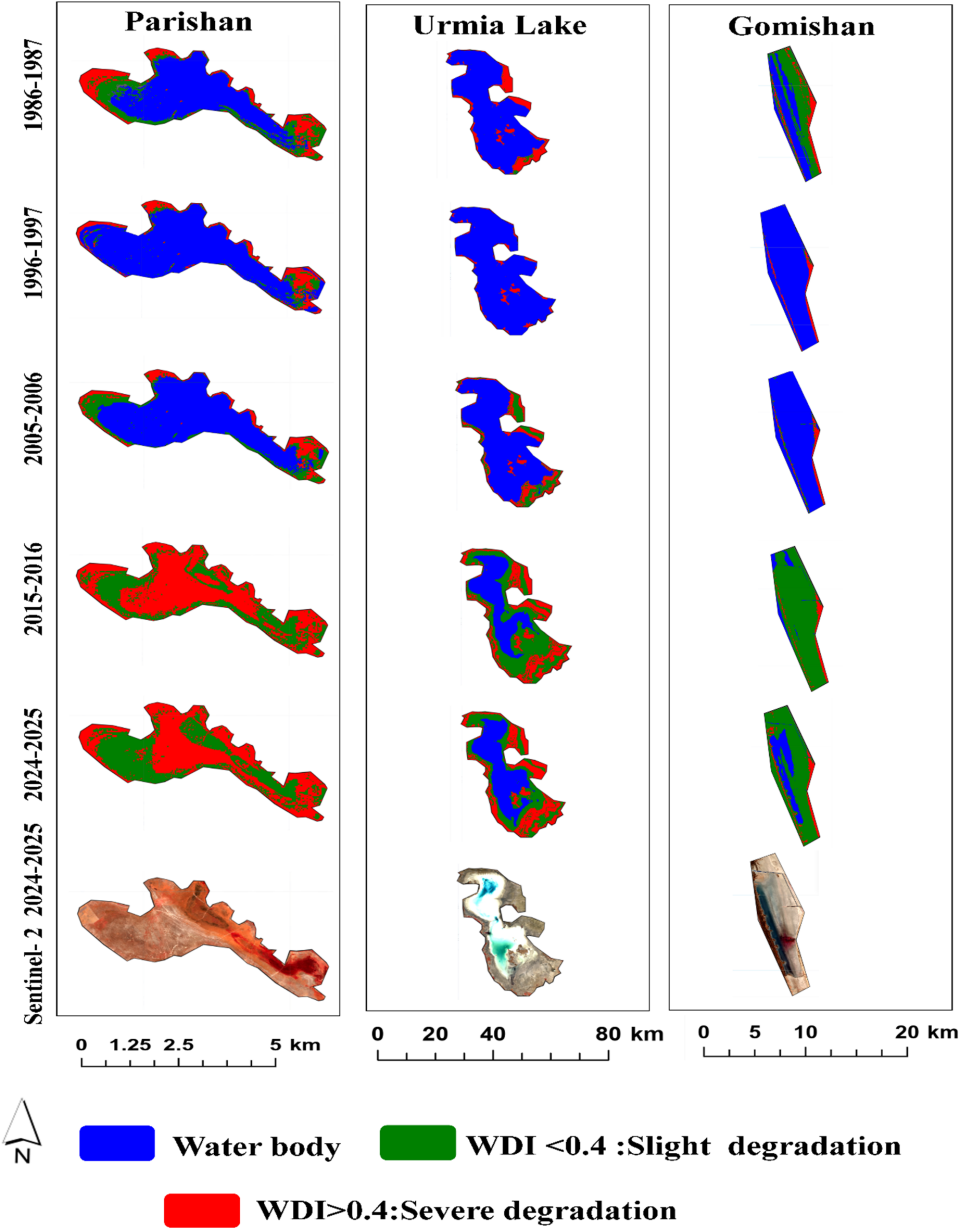
Spatial changes in slightly and severely degraded areas of critical international in Iran (1986–2024).

**Fig. 8:**
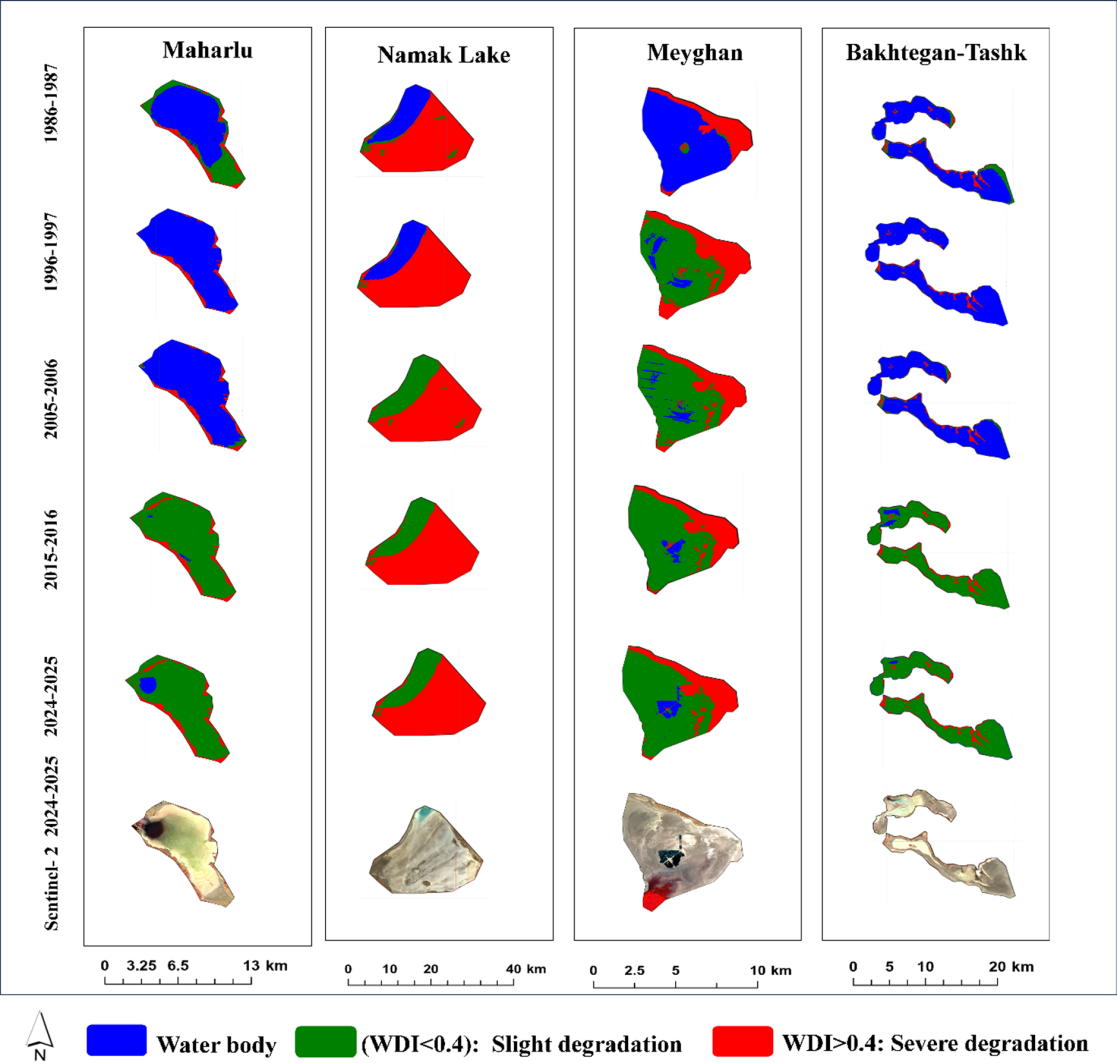
Spatial changes in slightly and severely degraded areas of critical national LWs in Iran (1986–2024).

Comparing the mean SDB area for LWs before and after the change point did not reveal a significant difference for the three wetlands: Shadegan, Hamun-e-Saberi, and Hamun-e-Puzak (P-Value > 0.05; Fig. 6). However, a significant difference in the mean SDB area was confirmed for the other seven wetlands (P-value < 0.05; Fig. 6). The spatiotemporal variations in the dried surface of these critical wetlands are presented in the following (Figs. 7 and 8).

The results indicate that Lake Parishan was in good condition during the first two decades of the study (1986–2006). Subsequently, the severity of degradation increased, leading to it completely drying up in 2015, and it has not yet recovered. Although the condition of Lake Urmia was better compared to other international LWs during the study period, it experienced the highest intensity of degradation at the beginning of the third decade (2015–2016) but has experienced a better situation in the fourth decade. Gomishan Wetland had a more stable condition from 1986 to 2006 compared to the years after that; however, its water-filled area has since decreased sharply by about 70%, and the extent of its degraded lands has increased (Fig. 7).

Maharlu and Bakhtegan-Tashk wetlands were in very favorable conditions during the first two decades of the study (1986–2006). Nevertheless, the water level gradually decreased, and their degradation level increased thereafter. Namak Lake, which was only water-filled in the northwestern areas during the period 1986–1996, has also been degraded and is classified as having low degradation in those areas. However, the intensity of degradation in other areas of the lake has been severe throughout the entire time period. More than 80% of Meyghan Wetland was water-filled in 1986–1987; in contrast, it gradually dried up after that, and now only a small part of the central areas of the wetland remains water-filled (Fig. 8). Sentinel-2 images also confirm the unfavorable conditions of these wetlands in the years 2024–2025.

In the next section, the contribution of the factors influencing the acceleration of degradation for these seven LWs was determined using RR before and after the breakpoint.

### Key influencing factors the LWs degradation in Iran

This study investigated the impact of fourteen key factors influencing the degradation of seven critical lake-wetlands. The mean values of all factors during the dusty season (April to September) and the average precipitation and runoff during the winter season were extracted for a 39-year period from Landsat data, TerraClimate products, and ERA5. Seasonal variations of all study variables during dusty periods are provided in the Supplementary Information (Figs. S2–S8). Winter variations in precipitation and runoff are shown in Fig. S9.

As explained earlier, VIF analysis was performed prior to the RR analysis to remove highly correlated independent variables. The results showed that most of the influencing variables are at an acceptable level (VIF < 4; Table S2). Some of them, such as RO for Meyghan, or PDSI for all selected LWs were identified as problematic variables.

After this step, Spearman correlation analysis was also performed to ensure that highly correlated variables were removed. The results showed that AirT variable had a strong correlation with VP in Bakhtegan-Tashk befor the break point (*p* = 0.74; Fig. S1); therefore, this variable was removed from the analysis and the modeling process was performed using the remaining variables.

**Fig. 9:**
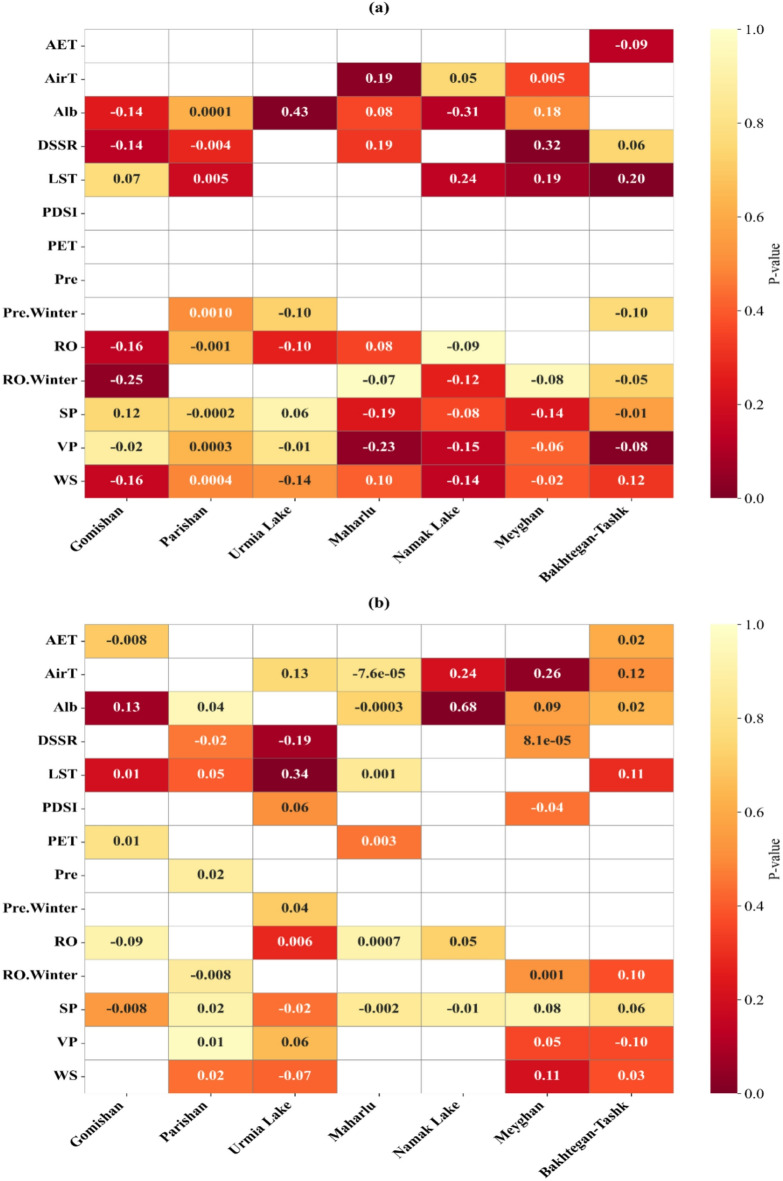
Relative importance of key influencing factors on the degradation of critical wetlands (**a**) before and (**b**) after the change point. Variables include AirT (air temperature), LST (land surface temperature), Pre (precipitation), WS (wind speed), AET (actual evapotranspiration), PET (potential evapotranspiration), RO (runoff), DSSR (downward surface shortwave radiation), VP (vapor pressure), SP (surface pressure), PDSI (Palmer Drought Severity Index), and Alb (albedo) during the dusty season. Pre.Winter and RO.Winter represent winter precipitation and winter runoff, respectively.

In the period before the change point; a decrease in RO.Winter (β= -0.25) was significantly associated with an increase in the SDB area of the Gomishan wetland (Fig. 9a). The most important driving factors for the desiccation of Urmia Lake and Meyghan were Alb (β = 043) and DSSR (β = 0.32), respectively. Two parameters of AirT (β = 0.19) and VP (β= -0.22) had a greater impact compared to other environmental drivers on the increasing trend of degradation in Maharlu. The desiccation of Bakhtegan-Tashk was associated with a significant increase in LST (β = 0.20) and a significant decrease in VP (β= -0.08).

In the period after the change point (Fig. 9b); the role of climatic factors has weakened. LST (β = 0.34) had the greatest impact on the severity of degradation in Urmia Lake. Furthermore, a significant increase in Alb(β = 0.68) and AirT (β = 0.26) was associated with the increased degraded area of Namak Lake and Meyghan wetland, respectively. For the other water bodies, the influence of the examined driving forces on desiccation was not statistically significant, a result which may indicate the dominance of management factors during this period.

**Fig. 10:**
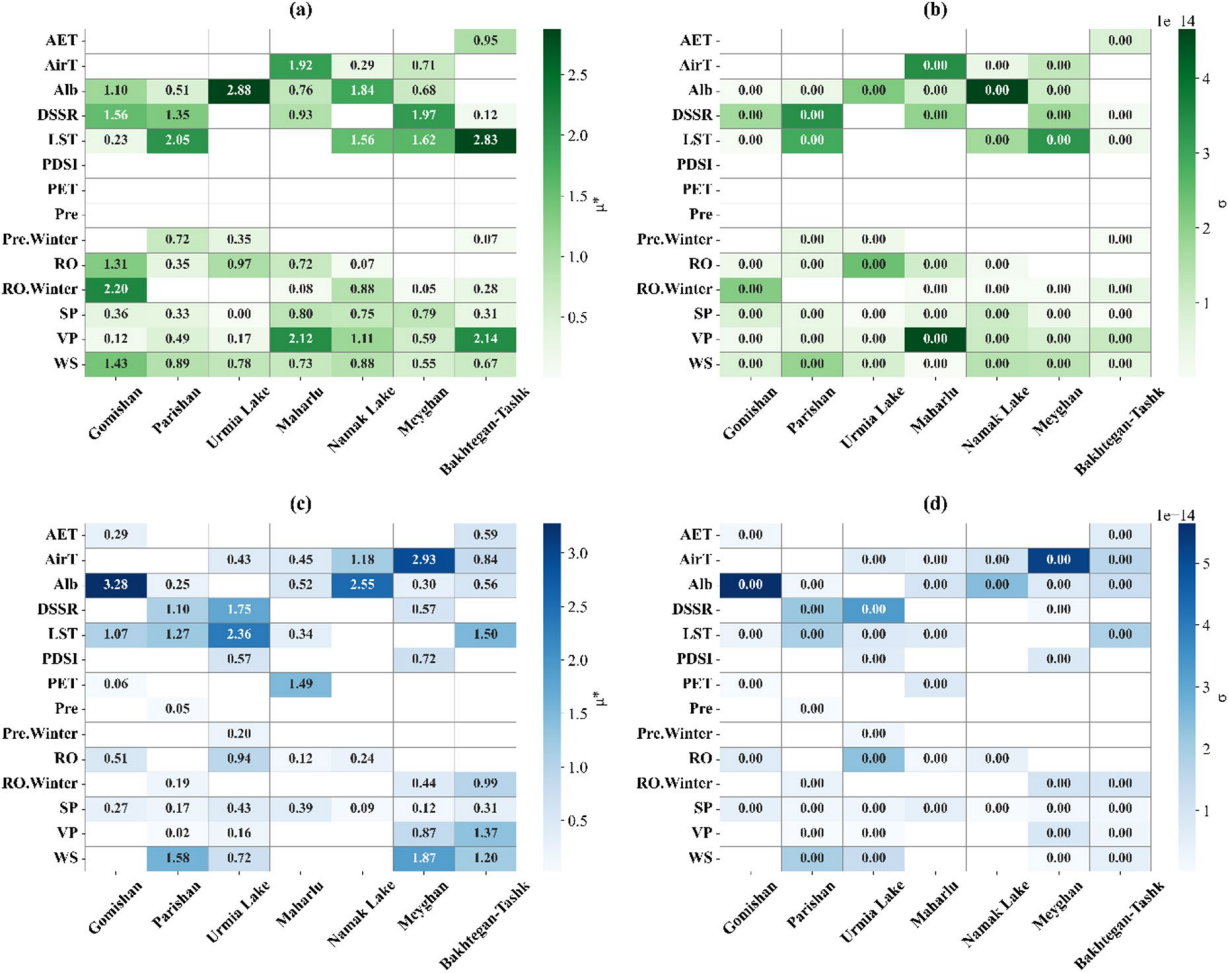
Results of the Morris sensitivity analysis for factors influencing the wetland degradation area before (**a**,**c**) and after (**b**,**d**) the change point. Variables include AirT (air temperature), LST (land surface temperature), Pre (precipitation), WS (wind speed), AET (actual evapotranspiration), PET (potential evapotranspiration), RO (runoff), DSSR (downward surface shortwave radiation), VP (vapor pressure), SP (surface pressure), PDSI (Palmer Drought Severity Index), and Alb (albedo) during the dusty season. Pre.Winter and RO.Winter represent winter precipitation and winter runoff, respectively.

The results of the Morris Sensitivity Analysis confirmed the findings obtained from the RR regarding the high importance of most influencing variables (Fig. 10). For example, the highest importance values before the change point for Urmia Lake and Meyghan were, respectively, Alb (µ = 2.87) and DSSR (µ = 1.97) (Fig. 10a). After the change point, LST (µ = 2.36), and AirT (µ = 2.93) were the most important variables for mentioned water bodies (Fig. 10c), which matched the results presented for the RR.

However, the results regarding the Morris sigma statistic (σ) showed that all values are very close to zero (Fig. 10b and d). This result is likely due to the small size of the datasets used before and after the change point, which is considered one of the limitations of the present study. Therefore, the Morris results should be interpreted with caution, and the RR estimates remain the most reliable representation of the relationships. Consequently, we rely on the estimates obtained from the RR method as the best statistical estimate of the relationships in this study.

In the current study, the RR model was chosen for use because the statistical periods before and after the change point were short, and the environmental factors could pose multicollinearity. A weakness inherent in the RR model is the fact that the model focuses on the linear association between the predictor and the dependent variable. While the Morris Method was used to improve on this weakness and establish whether any non-linear association exists, future studies should use alternative models that are more adept at modeling non-linear phenomena.

**Fig. 11:**
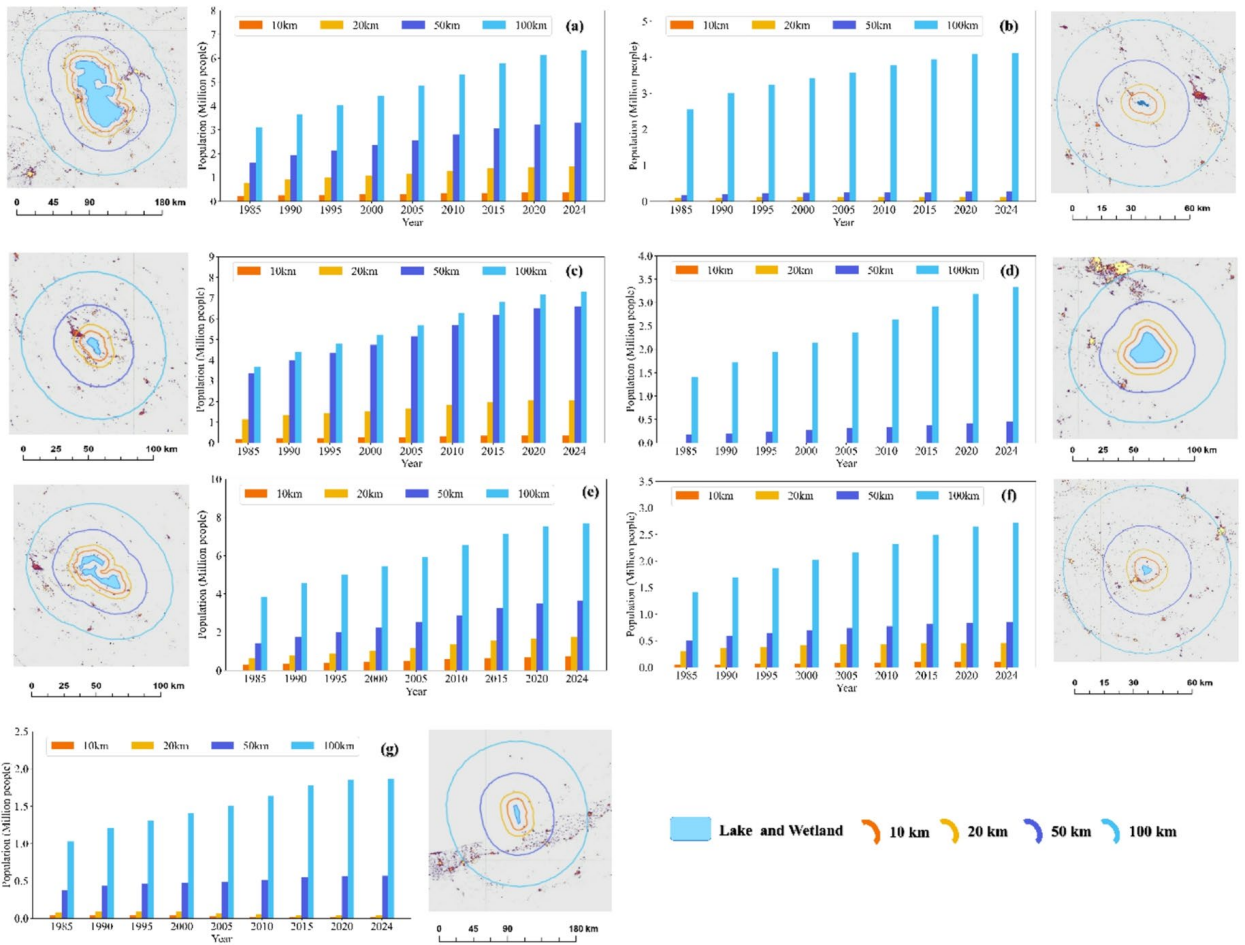
Spatial and demographic patterns around (**a**): Urmia Lake; (**b**) Parishan, (**c**) Maharlu, (**d**) Namak Lake, (**e**) Meyghan, (**f**) Bakhtegan-Tashk, and (**g**) Gomishan during 1985–2024.

Examination of population dynamics near critical LWs indicated population growth in all buffer zones around Lake Urmia, consistent with its expanding surface. While Parishan Wetland has a smaller surrounding population than Lake Urmia, growth is evident in all its buffer zones. Notably, population growth around Maharlu and Bakhtegan-Tashk is more pronounced in the 50–100 km range. Lake Namak exhibits the largest populations beyond 50 km, with an increasing trend observed over the study period. In 1985, the population within a 100 km radius of Lake Urmia and Lake Namak was roughly 3 and 1.5 million, increasing to about 6 and 3 million by 2024. Over the same period, populations around Parishan, Meyghan, Maharlu, Gomishan, and Bakhtegan-Tashk Wetlands grew from approximately 2.5, 1.5, 3.7, 1.2, and 3.8 million to about 4, 2.7, 7.3, 1.8, and 7.6 million, respectively (Fig. 11).

The results obtained from the Pearson correlation analysis indicated that there is no significant relationship between changes in population growth rates and changes in the SDB of critical wetlands and lakes. This finding suggests that mere population changes within the examined buffer zones were not the main determining factor for the intensity of wetland degradation during the study period.

However, despite the lack of a significant relationship, a positive correlation was observed between the changes in the SDB and changes in population growth rates in all different buffers surrounding the Bakhtegan-Tashk and Maharlu wetlands, and in the 10 to 50 km buffers of the Meyghan wetland and Urmia Lake (Table 3).

In contrast, this correlation was negative in the Gomishan and Parishan wetlands. This negative relationship could suggest the influence of other dominant factors, such as the volume of water extraction from surrounding wells and changes in land use, which occurred simultaneously with population growth and whose impact on degradation was stronger than the local population pressure.

**Table 3:** Correlation coefficients (r) between population growth rate in various buffers (10 to 100 km) and changes in severely degraded wetland area.

Wetland/Lake	10 km	*P*-value	20 km	*P*-value	50 km	*P*-value	100 km	*P*-value
Bakhteghan-Tashk	0.53	0.18	0.54	0.17	0.58	0.14	0.40	0.33
Gomishan	-0.60	0.12	-0.58	0.14	-0.44	0.27	-0.16	0.70
Maharlu	0.53	0.18	0.35	0.40	0.31	0.46	0.32	0.44
Meyghan	-0.14	0.73	0.34	0.42	0.38	0.36	0.36	0.38
Namak Lake	0.00	1.00	0.00	1.00	0.39	0.34	0.36	0.38
Parishan	0.00	1.00	-0.49	0.22	-0.46	0.25	-0.02	0.96
Urmia Lake	-0.11	0.79	0.02	0.96	0.07	0.87	0.08	0.86

### The impact of the severe degraded area in critical LWs on DEF

The annual trends of DEF versus the annual changes in severe degradation area in the critical LWs are shown in Fig. 12. It is noteworthy that this correlation was examined for the overlapping time period between the SDB extracted from Landsat images and the DEF calculated based on MODIS images (2000–2024).

**Fig. 12:**
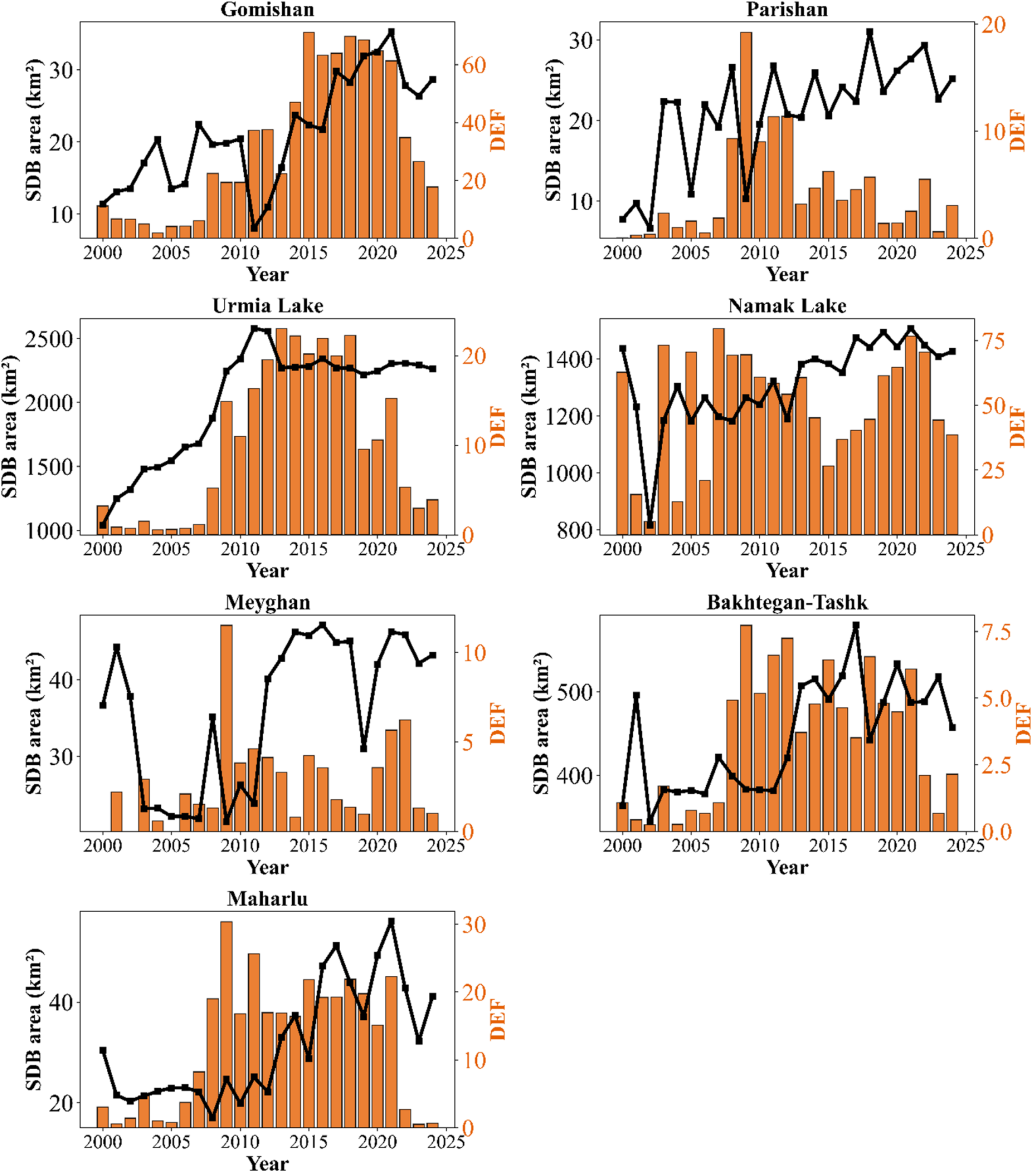
Yearly variations in dust event frequency and the extent of severe degradation (WDI > 0.4) in the study wetlands.

The results indicated that in this period, the long-term average DEF in Gomishan, Namak Lake, Maharlu, Urmia Lake, Parishan and Meyghan were 532, 1720, 699, 583, 248, and 71 events, respectively. Although significant intra-annual fluctuations in DEF are observed in the studied LWs, the peak dust activities in Gomishan wetland, Lake Urmia, Bakhtegan-Tashk, and Maharlu Lake is more evident in the years 2010 to 2020 compared to other studied years (Fig. 12).

**Fig. 13:**
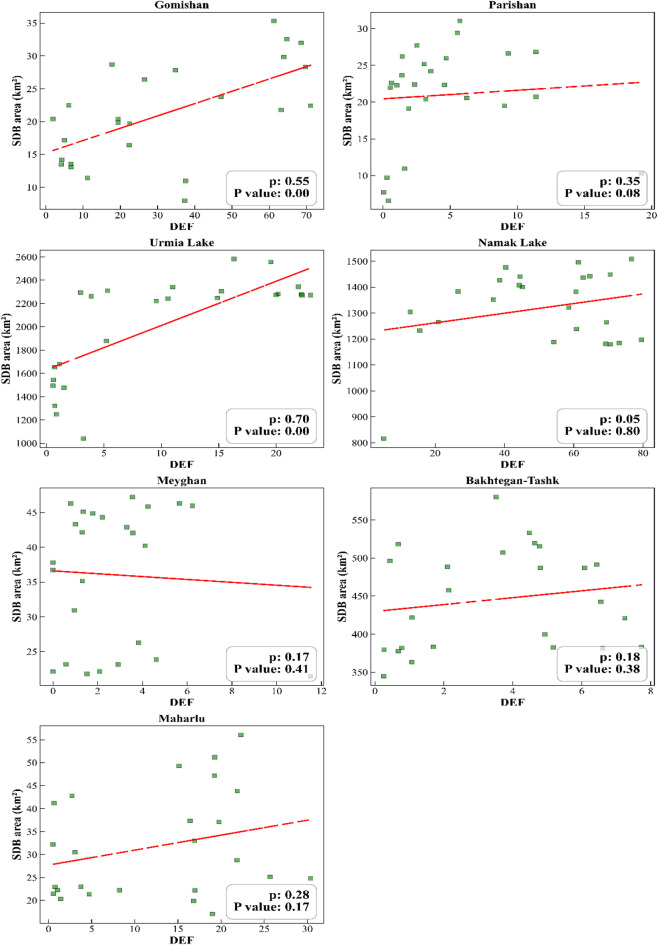
Pearson correlation of severely degraded wetland area with dust event frequency in critical Iranian lakes and wetlands (2000–2024).

Given the non-normal distribution of the data for the SDB area and/or the DEF (P-Shapiro-Wilk < 0.05; Table S3); the Spearman correlation coefficient was used to examine the relationship between them. The results showed that during the statistical period, there was a significant positive relationship between the SDB area of Gomishan, Parishan, and Urmia Lake with the DEF. The correlation coefficients obtained between these two variables in the mentioned LWs were estimated to be 0.55, 0.35, and 0.70, respectively. The relationship between SDB and DEF in Namak Lake (*p* = 0.05), Maharlu (*p* = 0.28), Meyghan (*p* = 0.17), and Bakhtegan-Tashk (*p* = 0.18) was also positive, which was statistically non-significant at the 5% level (P-value > 0.05) (Fig. 13).

Overall, considering the determination of the coefficient (R²) for Gomishan, Parishan, and Lake Urmi which had a significant impact on DEF, it can be concluded that approximately 30%, 12%, and 49% of dust events, respectively, were attributable to changes in the SDB of these water bodies over the past forty years.

## Discussion

### Spatiotemporal changes in severely degraded areas of LWs in Iran

Constant monitoring of the spatiotemporal changes of LWs by various instruments and methods is a major step towards the sustainable management of these valuable ecosystems^81,82^. One such tool is the WDI, which was used in this study to examine the beds of national and international importance of LWs of Iran. Over the past decades, these aquatic ecosystems have experienced drastic intra-annual fluctuations, which have led to fluctuations in the magnitude and intensity of their degradation^8^. Studies conducted on the trend of degraded area changes for 30 wetlands located in Iran showed that over the past 39 years (1986–2024), seven LWs had a significant increasing trend in the SDB area. Other wetlands showed a decreasing trend or no trend. A significant decreasing trend was observed only in the Delta-Rud-e-Gaz-Haraz and Khuran wetlands. Khanfari et al.^83^ reported that from 1980 to 2020, the vegetation cover of Shadegan wetland decreased and was converted to bare land. The results of these researchers are consistent with the increasing trend of the severe class in the present study. However, minor discrepancies may be due to differences in monitoring methodology (vegetation change in Khanfari and WDI in the present study) and the length of the time periods investigated.

The water level of Gomishan wetland showed an increasing trend in the period 1987–1998, followed by a sharp decrease. In this international wetland, fluctuations in the level of degraded lands have been high, and overall, the extent of its degraded lands has increased in the period 1986 to 2017^84^, which to some extent confirms our findings regarding the intensification of degradation in this wetland.

Research conducted by Rahimi et al.^85^ on 20 international wetlands of Iran for the period 2000–2020 using the maximum likelihood classification method showed that during this period, the water level of the delta-Rud-e-Gaz and Rud-e-Harra, the deltas-Rud-e-Shur, Rud-e-Shirin, and Rud-e-Minab, Kanibarazan wetland, and the Shurgol-Yadegarlu-Dorgeh increased by an average of about 8%. In contrast, it decreased by an average of about 20% in 11 wetlands. The largest decreases occurred in Gomishan, Alagol, Ulmagol, and Aghgol lakes, Parishan, and Lake Urmia. Although the method used and the study period of these researchers differ from the present study, the results are largely consistent.

Amindin et al.^30^, using the Mann-Kendall test, showed that the trend of changes in the water bodies of 27 international wetlands of Iran, except for Bakhtegan wetland, was decreasing in the statistical period 1990–2022. Although similar results were obtained for most of the studied wetlands, they do not match the results obtained for Bakhtegan wetland. In the present study, wetland monitoring was conducted based on changes in the beds of severely dried wetlands over a 40-year statistical period, whereas in Amindin’s study, monitoring was evaluated based on changes in the surface area of the wetlands’ water bodies over a 32-year period. This difference in the chosen indicator and the length of the statistical period could be the main reason for the discrepancy in results for Bakhtegan Wetland. However, it is consistent with the findings of Mozafari et al.^5^ regarding the intensification of degradation in this international wetland.

Identifying the spatiotemporal changes of water bodies and understanding their degradation status is a primary step in pinpointing critical LWs. However, recognizing the key factors driving the intensification of degradation in these ecosystems is also crucial for mitigating their adverse consequences. Given that seven LWs in Iran are currently severely degraded, the next section will discuss the key influencing factors.

### Key factors affecting the intensification of LWs degradation in Iran

Wetland degradation is typically caused by the complex interaction of numerous climatic, terrestrial, and human activities^55,86^. Given that attempting to solve all problems and control all influencing factors simultaneously is not feasible, focusing on identifying the most critical factors can be more effective for management and executive actions. Numerous factors influence the LWs degradation^87,88^. However, the extent of each factor’s impact varies among different ecosystems, especially in wetlands^89^.

The findings of this study indicated that during the pre-change period, the relative contribution of climatic factors to the intensity of LWs degradation was greater than that of other influencing factors. Increased AirT and a decrease in VP were the main drivers of degradation in the Maharlu Wetland in southwestern Iran, a condition that likely led to intensified evaporation and a disruption of the surface energy balance. In Bakhtegan-Tashk, decreased VP accompanied by an increase in LST were associated with the intensification of the contraction of this aquatic ecosystem. This demonstrates the role of thermal processes and changes in surface energy exchange in the increasing trend of their severely degraded beds. With an increase in LST, the rate of evaporation increases and soil moisture decreases, leading to desiccation and the expansion of the degraded surface area^90^. LST is representative of land use changes^91^and its impact on the desiccation of aquatic ecosystems has been proven by Rezapouraghdam et al.^92^, which supports the findings of the present study .

Surface Alb is a function of changes in several factors such as vegetation cover, water level, salinity, and the type and color of soil sediments^93–96^. Increased Alb leads to increased reflection from the Earth’s surface, which can decrease temperature and potentially mitigate wetland degradation^97^. However, the present study suggests that albedo may also be associated with increased degradation. Generally, these findings highlight the dual role of albedo as both a potential consequence of wetland degradation and, when considered as an input variable, a contributing factor to it.

As has been proven in the present study and previous studies^78,98^, the volume and depth of Lake Urmia’s water have decreased in recent years. Given that shallow waters are more affected by air temperature and evaporation occurs more rapidly in them^99^, the lake’s degradation has intensified. Another major factor in the lake’s degradation was identified as the increased Alb of the lake’s surface. Lake Urmia is one of the largest saline lakes in Iran, and its salt surface area has increased in recent years, impacting the increase of its albedo^100^. For this reason, its key role in the lake’s degradation has been highlighted in this study.

In the post-change period, increased LST, Alb, and AirT were the only significant factor influencing Lake Urmia, Namak Lake, and Meyghan, respectively, which likely indicates the key role of managerial and anthropogenic factors during this timeframe.

In this study, the trend of population change around the seven identified critical wetlands was increasing; however, statistically, no significant meaningful relationship was observed with the trend of changes in the extent of wetland degradation. One possible reason may be related to examining this relationship using datasets with five-year temporal intervals. Nevertheless, this pattern may indicate the potential role of human activities in the degradation of these areas. This is because population growth leads to increased demand for the exploitation of surface and groundwater resources for agricultural development, urbanization, and industry, ultimately reducing the water level of the wetlands and lakes and accelerating the degradation process^4,101^. Expanding degraded land area has also been confirmed in previous studies, corroborating the findings of our research^102,103^.

Although the present study provided a more comprehensive view of the factors affecting wetland degradation by using a 40-year dataset on the degraded area of wetlands to analyze the degradation trend of the dried-up surface of 30 important national and international wetlands in Iran, identify change points, effective climatic and terrestrial factors on these changes in critical wetlands, and investigate the role of population dynamics, it had several limitations:

First, insufficient access to annual information regarding land use changes, the number of dams constructed upstream of the wetlands, and the volume of water withdrawal from surface and groundwater sources, and the lack of their inclusion as key human-centric parameters in the modeling.

Second, the variable quality of Landsat data over the study period may introduce inconsistencies in detecting changes in severely degraded wetland beds. Third, the short length of the statistical sub-periods, which limited the use of complex non-linear models for modeling and analyzing the contribution of influential factors. Fourth, the inability of Morris sensitivity analysis to discover non-linear relationships. Therefore, it is suggested that future studies be conducted with a focus on addressing the mentioned limitations to provide a more comprehensive view of the wetland degradation trend and the influencing factors.

One of the main consequences of degradation, especially in severely damaged wetlands and lakes, is the increased emission of dust due to the heightened sensitivity of the bed of these valuable ecosystems^104,105^. The impact of the shrinkage of critical Iranian wetlands and lakes on this environmental hazard is discussed in the subsequent section.

### Impact of critical LWs degradation on dust event frequency

In this study, it was demonstrated that the degraded area of the Gomishan International Wetland on the Iran-Turkmenistan border and Lake Urmia in northwestern Iran have had the most significant impact on increasing the DEF and dust pollution. The examination of the spatial changes in the dried-up bed of these water bodies revealed that the eastern and southern regions of Lake Urmia have been severely degraded, leading to the expansion of desert areas in this part, which aligns with the findings of Ghale et al.^106^, regarding the degradation of this part of the lake and the increased occurrence of dust in northwestern Iran, as also substantiated in other studies^78,107,108^.

Our findings revealed that despite the increasing trend in the severely degraded part of Meyghan wetland, the frequency of dust events has decreased. This may be attributed to the changing due to the proportion of salts in the dust for different dried surfaces or the formation of a hard crust on the wetland’s surface that prevents the emission of dust particles^109–111^. Other hypotheses, such as stabilization efforts, changes in regional wind patterns and surrounding land uses, and a reduction in the intensity of storms capable of detaching wetland bed particles, are beyond the scope of this research and are recommended for investigation in future studies.

The three wetlands of Bakhtegan-Tashk, Maharlu, and Parishan in southwestern Iran have also played a crucial role in increasing the frequency of dust events and dust pollution in this region of Iran over the past four decades. Hamzeh et al.^78^ reported that during the statistical period of 2003 to 2017, the trend of aerosol optical depth changes over Lake Urmia and Bakhtegan was increasing and then decreased until 2020, which is consistent with the findings of this study within these timeframes. They attributed the likely cause of these changes to the desiccation of the bed of these two international wetlands and lakes, a finding supported by the present study.

In this study, it was determined that during the period of 2000–2024, on average, approximately 12% of the changes in DEF in the Parishan wetland was a function of the increasing in the SDB area. Ebrahimi-Khusfi et al.^31^ demonstrated that between 1980 and 2018, approximately 15% of the dust pollution around this lake, was due to the decrease in its water area, corroborating the findings of this study regarding the impact of the degradation of the lake on increasing the dust pollution. The reason for the difference in findings is the difference in the length of the statistical period studied and the type of criteria for measuring dust events. Furthermore, local topography and regional meteorology play a significant role in AOD variations, which may strongly influence the correlations.

Based on the findings of this study, it was also found that despite the small number of critical wetlands, their impact on intensifying dust events has been considerable. Approximately 12%, 30%, and 49% of the variations in DEF were attributable to changes in their SDB, respectively. In a study conducted by Ebrahimi-Khusfi et al.^112^, the contribution of the dried Meyghan wetland to the increase in dust events at the closest synoptic station to the wetland was reported to be about 23%. The difference in the role of various wetlands in intensifying dust events may be due to specific physical characteristics of their surfaces, such as the presence or absence of degraded crusts and soil texture as well as local wind patterns^43,113,114^.

In wetland environments, the dried bed consists of fine-grained sediments and unstable salt crusts which, upon exposure to wind, are highly susceptible to erosion and dust production^40,43^. In contrast, in natural dry environments, these phenomena primarily occur in coarser-grained soils that are adapted to the arid climate^115,116^. Furthermore, with the drying of wetland environments, hydrophytic plants, which are a factor in creating surface roughness and resistance to wind erosion, perish^117,118^. As a result, soils are more easily lifted by lower-speed winds, and the frequency of dust events increases. These results show that, besides the area of dried-up beds, the physical-chemical properties of the wetland surfaces and regional disparities in the composition of the soil and vegetation cover also have an influence on the extent and occurrence of the dust events. This may explain the discrepancies observed with previous research.

On a regional scale, increased emission of dust aerosols or intensified dust activity within the boundaries of northern China wetlands^89^, the Middle East^119^, and Central Asia^120^due to the degradation of these valuable aquatic ecosystems has been reported, which is consistent with the results of the present study.

The primary focus of this study was to analyze the role of dried-up Iranian LWs beds on the occurrence of domestic dust storms. The results confirmed the role of some of these wetlands in intensifying dust pollution in these areas. However, several limitations are mentioned here. For instance, excluding the influence of other climatic and terrestrial factors; land-use changes in upstream watersheds; possible errors resulting from the fixed threshold AOD > 0.5; the role of trans-regional and large-scale wind patterns; or human management interventions, such as water diversion and wetland restoration. In this regard, it is recommended that further research involves these effects, apart from the impact of dried wetland beds, to grasp the full comprehension of all driving forces.

Although the findings of this study indicate that part of the increasing trend in dust event occurrence in recent years is associated with the expansion of dried areas of certain inland lakes and wetlands in Iran, this increasing trend cannot be attributed solely to the influence of these local factors. Large-scale atmospheric circulation patterns, land surface conditions in areas surrounding water bodies, changes in wind regimes, and the presence of active dust sources at regional and transboundary scales can significantly affect the intensity, frequency, and spatial distribution of dust events^38,121^. These factors may either independently influence dust activity or interact with lake desiccation processes to amplify or mitigate dust emissions^31^. Therefore, quantitatively determining the relative contribution of each of these factors to the occurrence and intensification of dust events, while highly important, is beyond the scope of the present study and can be considered a key topic for future research.

## Conclusions

Understanding the long-term trends of change in aquatic ecosystems, identifying their primary drivers, and analyzing their impact on dust activities, is of significant importance, particularly in Iran. The long-term trends in the SDB of 30 national and international LWs in Iran over the period 1986–2024 were investigated for the first time in this study using the WDI. Based on the findings of this study, it was determined that out of 30 water bodies in Iran, nine (approximately 30%) have exhibited a significant increasing trend in their SDB area over the last four decades (Z > + 2; P-value < 0.05). This result was confirmed for 7 of these water bodies (23%) using the Mann-Whitney U test and T-test for the periods before and after the change point at the 95% confidence level. This suggests that relying solely on the Mann-Kendall test results is insufficient for analyzing the trend of changes in the desiccated wetland bed and confirms the necessity of implementing complementary tests. Climatic and land-based factors were identified as the main driving forces for these changes before the change point. The diminished contribution of these factors in most critical wetlands, concurrent with an increase in population pressure, demonstrated the active role of human activities in the deterioration of the ecological conditions of some critical water bodies in Iran. Furthermore, it was determined that the desiccation of most critical LWs has led to an increase in air dustiness in these regions. Among them, the impact of desiccation in Gomishan, Parishan, and Urmia Lake was significantly greater than the impact of desiccation in Namak Lake, Bakhtegan-Tashk, and Maharlu. The correlation coefficient between SDB and DEF in the three most critical LWs was estimated to be 0.55, 0.35, and 0.70, respectively, indicating that approximately 30%, 12%, and 49% of the dust events were caused by their severe bed degradation. Changes in SDB showed a statistically significant contribution to DEF variability in several of the studied wetlands. Therefore, continuous monitoring of all aquatic ecosystems in Iran is essential, particularly the 23% that have experienced an accelerating trend of degradation. The lack of access to annual information on activities such as land-use changes, the number of dams constructed upstream of the wetlands, and the amount of water withdrawn from surface and groundwater sources are among the limitations of the present study. It is recommended that the annual impact of these driving factors, along with climatic and land-based factors, on the trend of changes in these valuable ecosystems be investigated in future studies. It is also suggested that the assessment of wetland degradation trends be examined with other composite indices to achieve a more comprehensive understanding of the trend of changes in different degradation classes.

## Supplementary Information

Below is the link to the electronic supplementary material.

Supplementary Material 1

## Data Availability

The datasets used and/or analyzed during the current study are available from the corresponding author on reasonable request.
