# Supplementary material for: Degradation trends in lakes and wetlands of Iran and their contribution to dust pollution
Source: Sci Rep. 2026 Feb 18;16:9503. doi: 10.1038/s41598-026-40357-1 (PMC13005010; doi:10.1038/s41598-026-40357-1)
Supplement: Supplementary file 1 — Supplementary Material 1 [file 41598_2026_40357_MOESM1_ESM.docx]

**Supplementary material**

**Table S1.** The applied software used in this study

| Software | Description | Source |
| --- | --- | --- |
| QGIS | Export Image | https://qgis.org/ |
| Google Earth Engine | Processing and extracting various indicators and parameters | https://earthengine.google.com/ |
| Jupyter notebook | Drawing diagrams | https://jupyter.org/ |
| statsmodels package | Variance Inflation Factor (VIF) | https://www.statsmodels.org/stable/index.html |
| scikit-learn package | Ridge Regression | https://scikit-learn.org/ |
| pymannkendall package | Mann-Kendall test | https://github.com/mmhs013/pymannkendall |
| Seaborn package | Heatmap Chart | https://seaborn.pydata.org/ |
|  | | |

**Table S2.** Variance inflation factor values for influencing factors the wetlands degradation in Iran.

| **Variable** | **Before change point** | | | | | |
| --- | --- | --- | --- | --- | --- | --- |
|  | **Gomishan** | **Parishan** | **Maharlu** | **Namak Lake** | **Meyghan** | **Bakhtegan-Tashk** |
| Alb | 1.4 | 2.0 | 1.8 | 2.9 | 3.5 | 1.8 |
| LST | 2.3 | 1.7 | 2.7 |  | 3.0 | 2.4 |
| RO | 2.1 | 1.7 | 1.9 | 1.8 | 1.7 |  |
| SP | 2.7 | 1.4 | 1.3 | 1.8 | 2.3 | 1.5 |
| DSSR | 2.8 | 2.0 |  | 1.9 |  | 2.2 |
| VP | 2.1 | 1.4 | 2.6 | 2.9 | 2.1 | 1.7 |
| WS | 1.9 | 2.4 | 1.8 | 1.9 | 1.6 | 1.5 |
| Pre.Winter | 2.0 | 1.7 | 1.2 | 3.9 | 2.1 |  |
| RO.Winter | 2.9 |  |  | 3.6 | 1.7 | 1.5 |
| AirT |  |  |  | 2.0 | 2.4 | 2.3 |
| AET |  |  |  |  |  |  |
|  | **After change point** | | | | | |
| AET | 1.2 |  |  |  |  |  |
| Alb | 2.5 | 2.4 | 3.7 | 1.6 | 1.0 | 2.4 |
| LST | 2.1 | 2.6 | 3.9 | 2.1 |  |  |
| PET | 1.3 |  |  | 1.3 |  |  |
| RO | 1.9 |  | 3.3 | 1.5 | 1.7 |  |
| SP | 1.6 | 1.5 | 1.6 | 1.9 | 1.4 | 1.4 |
| Pre |  | 2.2 |  |  |  |  |
| DSSR |  | 3.0 | 3.7 |  |  | 2.2 |
| VP |  | 1.3 | 1.9 |  |  | 2.5 |
| WS |  | 2.9 | 1.5 |  |  | 2.9 |
| RO. Winter |  | 1.3 |  |  |  | 2.0 |
| AirT |  |  | 2.1 | 1.5 | 1.4 | 3.0 |
| PDSI |  |  | 2.4 |  |  | 2.6 |
| Pre.Winter |  |  | 2.1 |  |  |  |


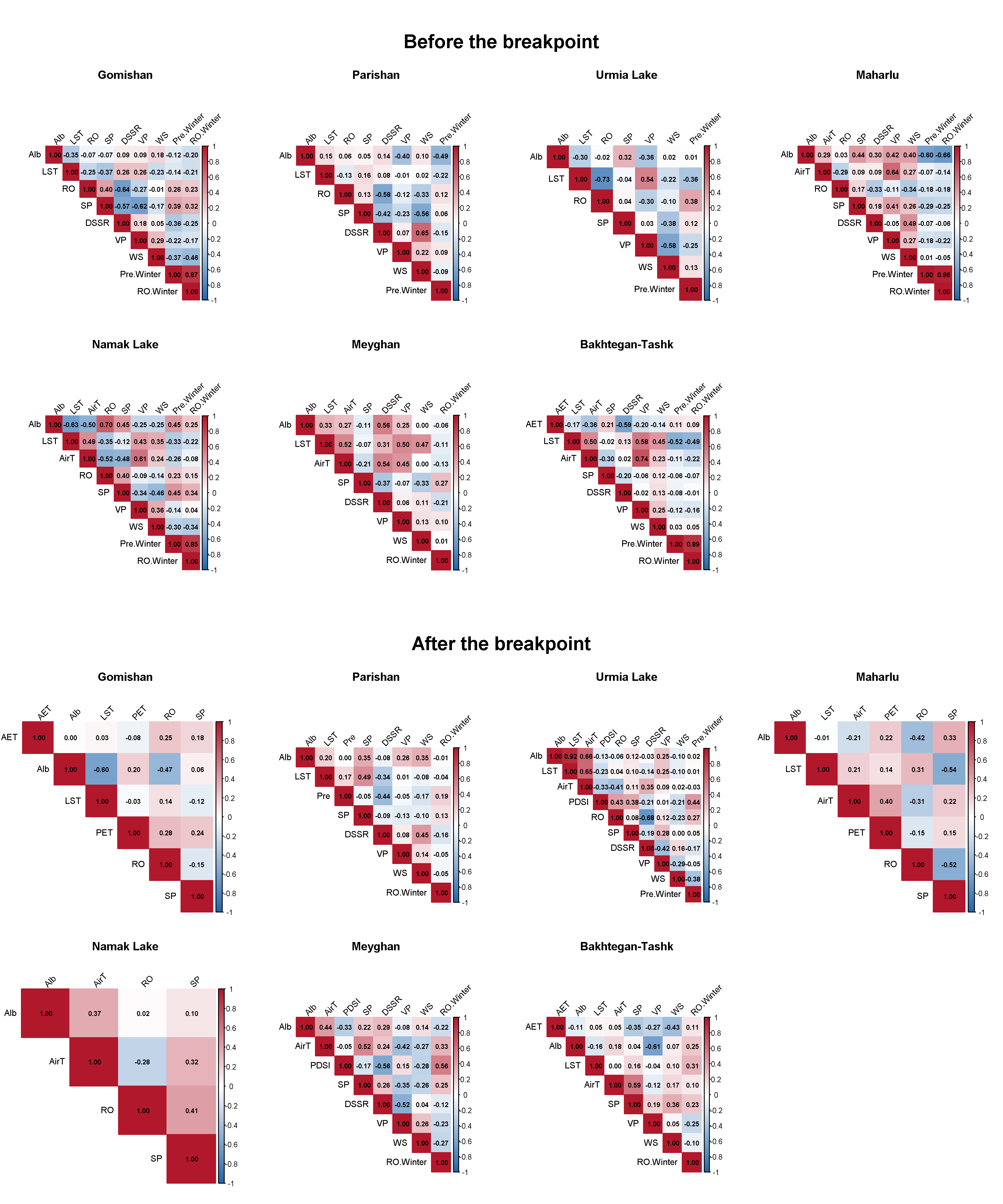


**Fig. S1.** Spearman correlation coefficients among variables influencing the degradation of critical wetlands, after assessing and removing multicollinearity (VIF).

**Table S3.** The results of the Shapiro-Wilk normality test for data related to the sever degraded beds (SDB) area and dust events frequency (DEF) in Iranian lakes and wetlands.

| **Variable** | **Lake and wetland** | **Test Statistic** | **P-value (Shapiro-Wilk)** |
| --- | --- | --- | --- |
| DEF | Gomishan | 0.87 | 0.00 |
|  | Parishan | 0.82 | 0.00 |
|  | Urmia Lake | 0.86 | 0.00 |
|  | Maharlu | 0.88 | 0.01 |
|  | Namak Lake | 0.93 | 0.07 |
|  | Meyghan | 0.84 | 0.00 |
|  | Bakhtegan-Tashk | 0.91 | 0.03 |
| SDB area | Gomishan | 0.97 | 0.66 |
|  | Parishan | 0.89 | 0.01 |
|  | Urmia Lake | 0.84 | 0.00 |
|  | Maharlu | 0.90 | 0.02 |
|  | Namak Lake | 0.87 | 0.00 |
|  | Meyghan | 0.83 | 0.00 |
|  | Bakhtegan-Tashk | 0.92 | 0.05 |


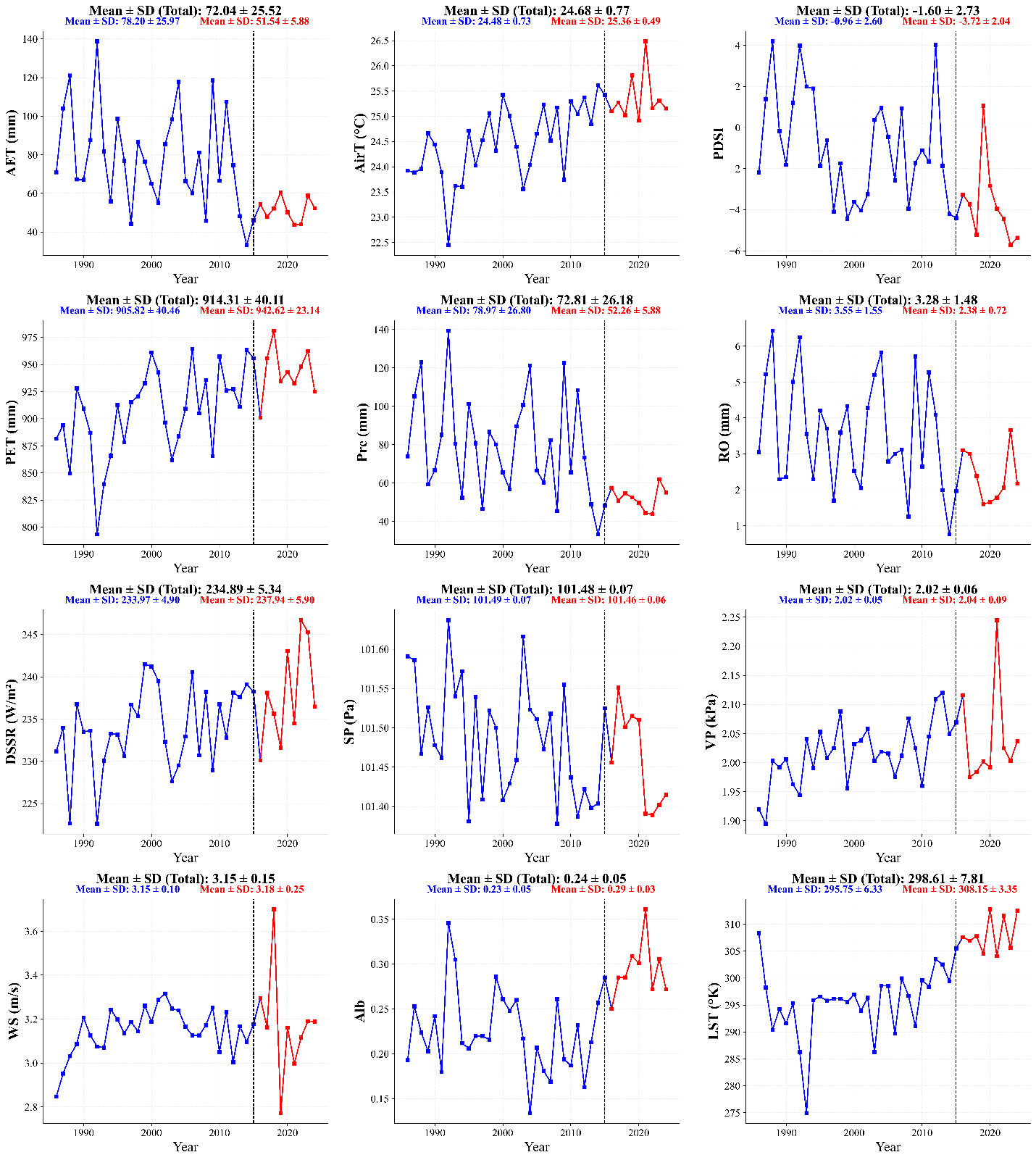


**Fig.S2. Seasonal changes during dusty seasons in AET (actual evapotranspiration), AirT (air temperature), Palmer drought severity index (PDSI), potential evapotranspiration (PET), precipitation (Pre), runoff (RO), Downward surface shortwave radiation (DSSR), surface pressure (SP), vapor pressure (VP), wind speed (WS), albedo (Alb), and land surface temperature (LST) surrounding Gomishan Wetland, Iran.** **The dashed vertical line in each panel indicates the statistically determined break point. Mean ± SD (Standard Deviation) for the total period, the pre-change, and the post-change point period are shown with black, blue and red colors, respectively.**


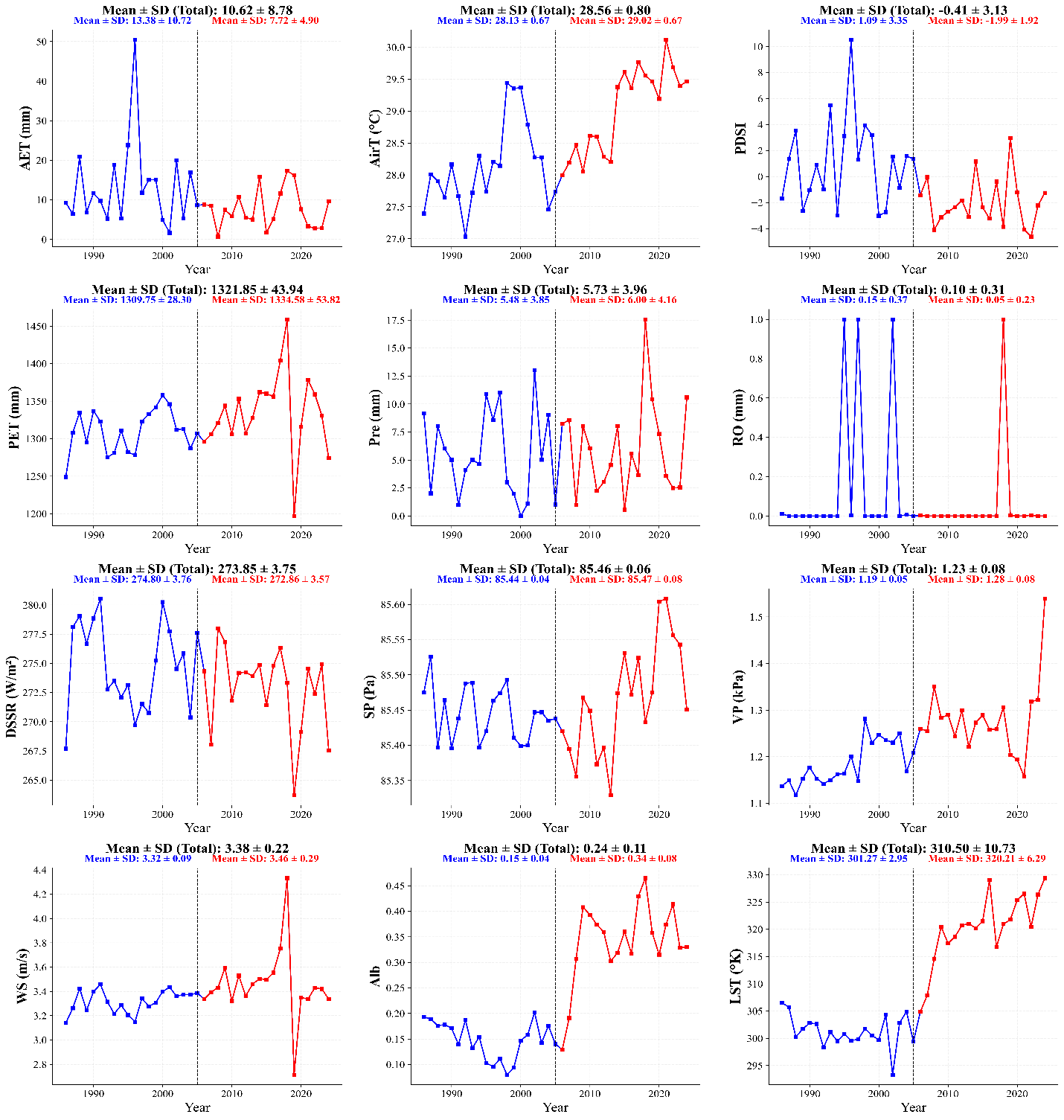


**Fig.S3. Seasonal changes during dusty seasons in AET (actual evapotranspiration), AirT (air temperature), Palmer drought severity index (PDSI), potential evapotranspiration (PET), precipitation (Pre), runoff (RO), Downward surface shortwave radiation (DSSR), surface pressure (SP), vapor pressure (VP), wind speed (WS), albedo (Alb), and land surface temperature (LST) surrounding Parishan Lake, Iran. The dashed vertical line in each panel indicates the statistically determined break point. Mean ± SD (Standard Deviation) for the total period, the pre-change, and the post-change point period are shown with black, blue and red colors, respectively.**


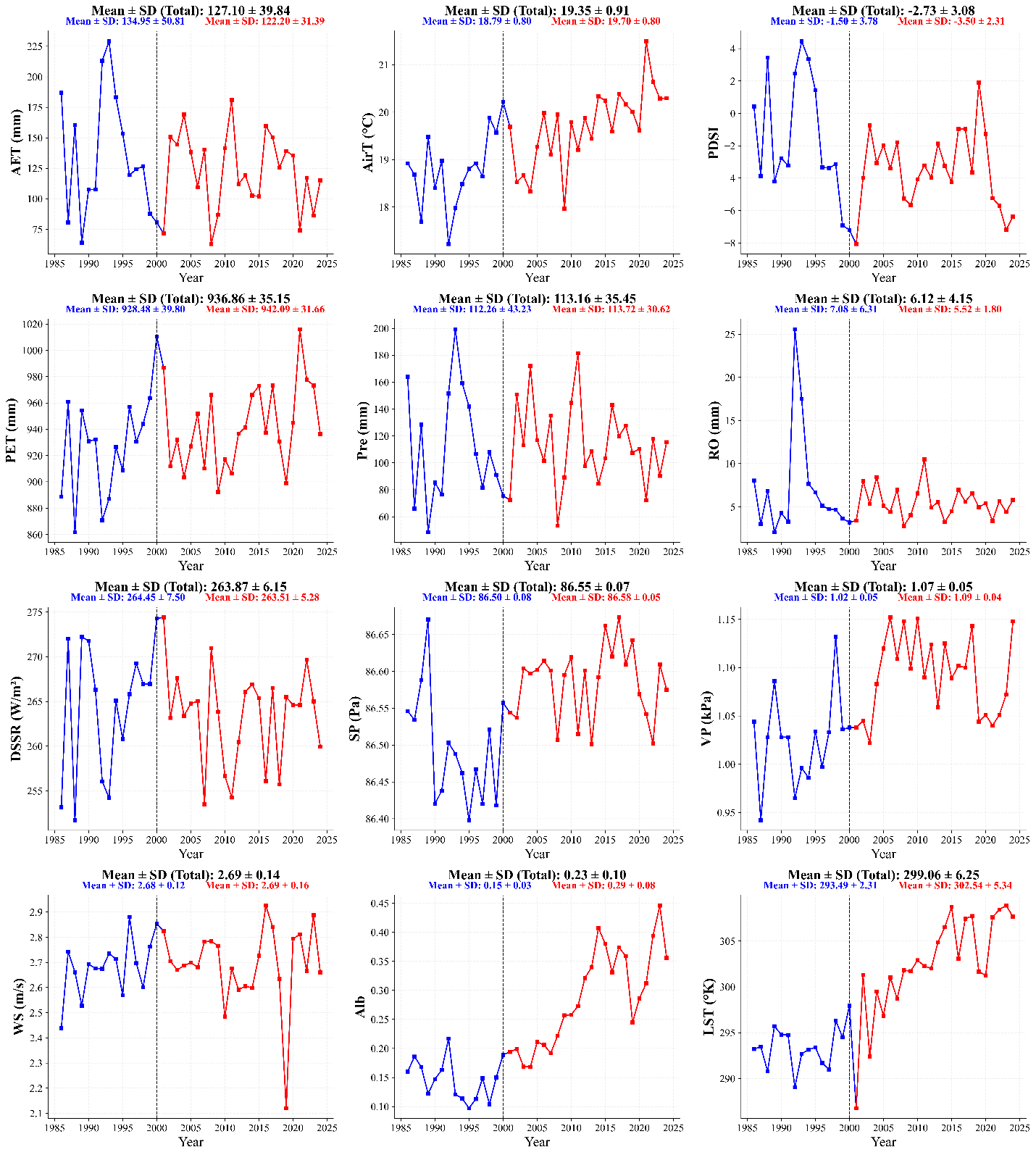


**Fig.S4. Seasonal changes during dusty seasons in AET (actual evapotranspiration), AirT (air temperature), Palmer drought severity index (PDSI), potential evapotranspiration (PET), precipitation (Pre), runoff (RO), Downward surface shortwave radiation (DSSR), surface pressure (SP), vapor pressure (VP), wind speed (WS), albedo (Alb), and land surface temperature (LST) surrounding Urmia Lake, Iran. The dashed vertical line in each panel indicates the statistically determined break point. Mean ± SD (Standard Deviation) for the total period, the pre-change, and the post-change point period are shown with black, blue and red colors, respectively.**

**
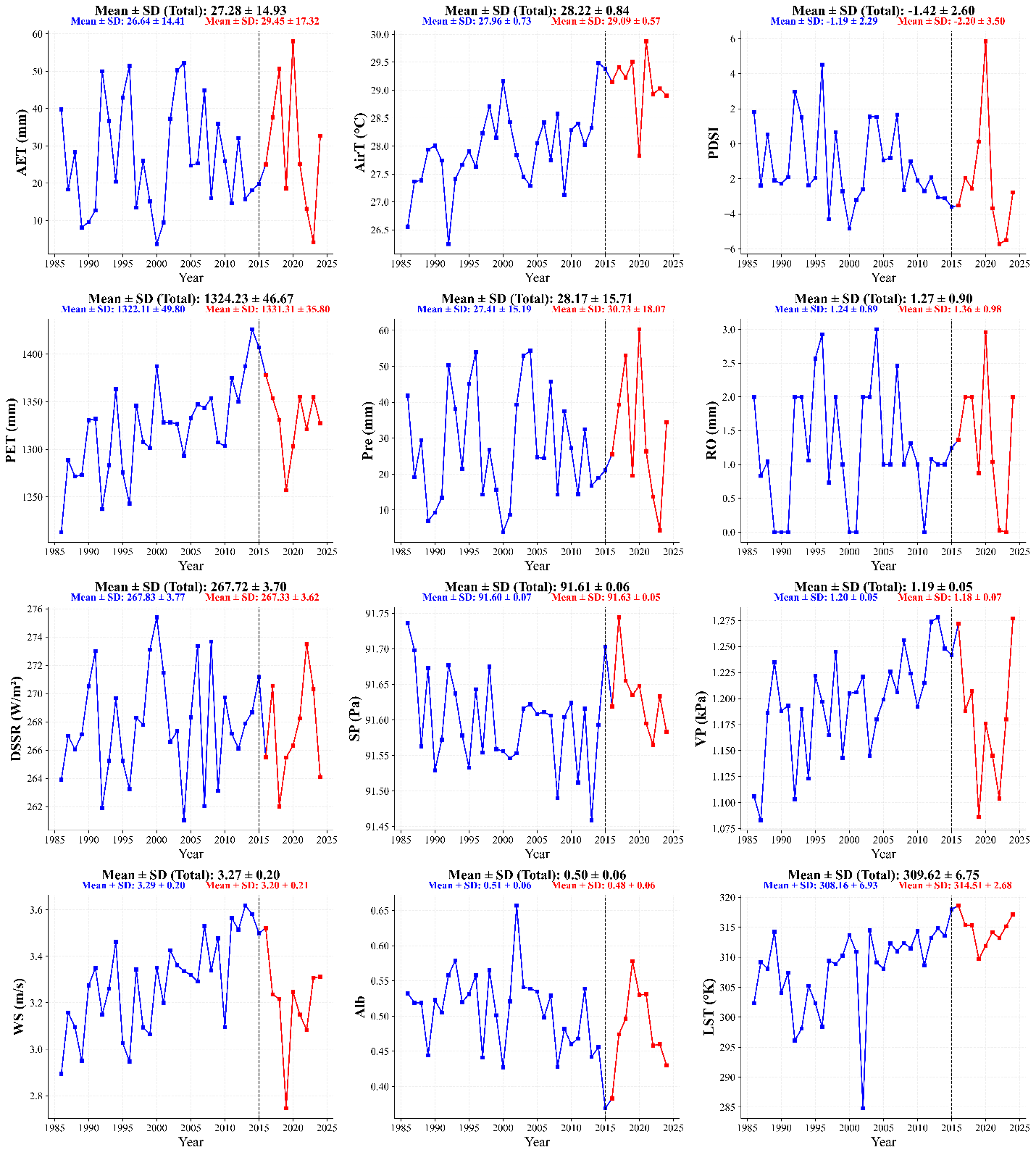
**

**Fig.S5. Seasonal changes during dusty seasons in AET (actual evapotranspiration), AirT (air temperature), Palmer drought severity index (PDSI), potential evapotranspiration (PET), precipitation (Pre), runoff (RO), Downward surface shortwave radiation (DSSR), surface pressure (SP), vapor pressure (VP), wind speed (WS), albedo (Alb), and land surface temperature (LST) surrounding Namak Lake, Iran. The dashed vertical line in each panel indicates the statistically determined break point. Mean ± SD (Standard Deviation) for the total period, the pre-change, and the post-change point period are shown with black, blue and red colors, respectively.**

**
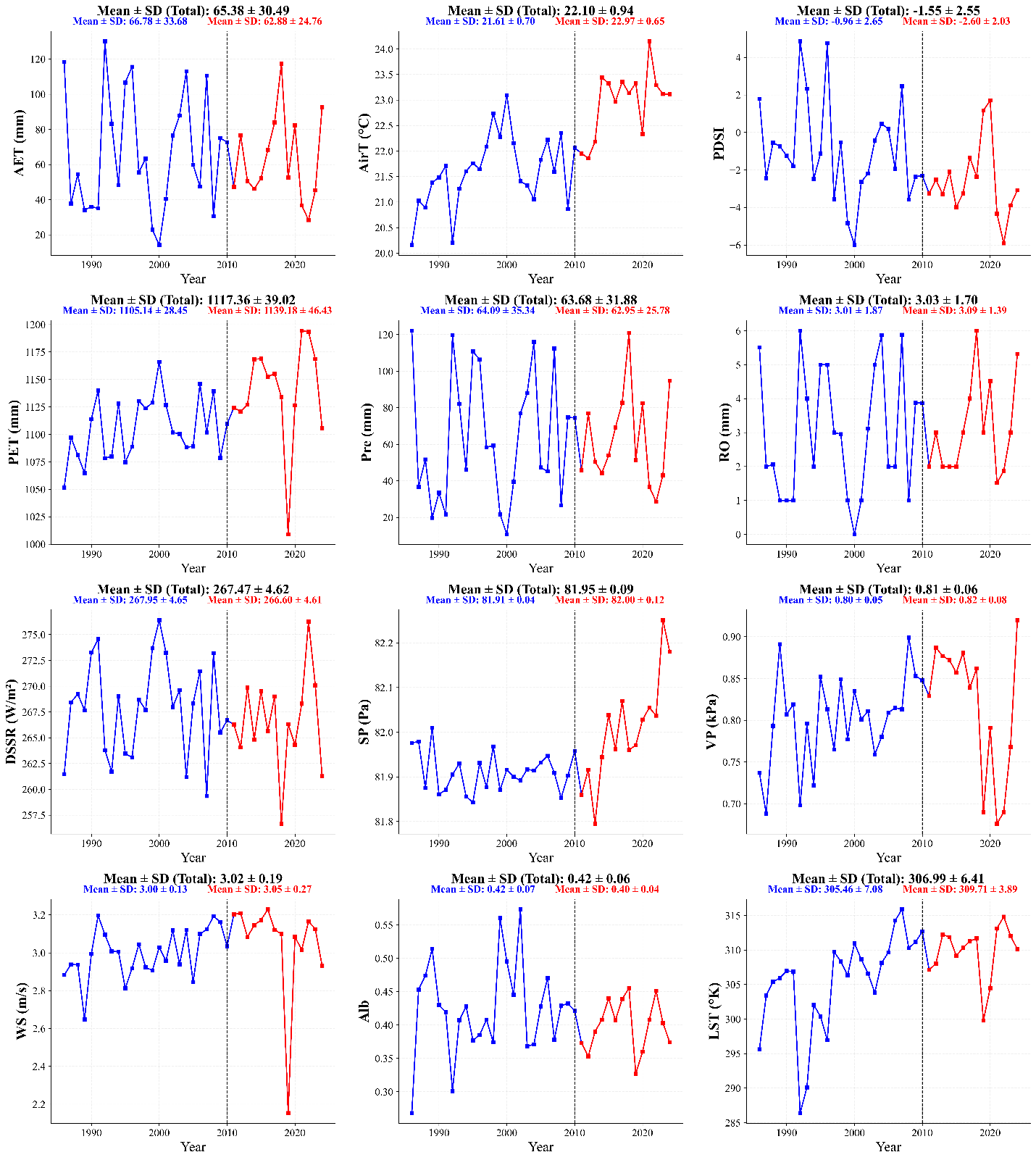
**

**Fig.S6. Seasonal changes during dusty seasons in AET (actual evapotranspiration), AirT (air temperature), Palmer drought severity index (PDSI), potential evapotranspiration (PET), precipitation (Pre), runoff (RO), Downward surface shortwave radiation (DSSR), surface pressure (SP), vapor pressure (VP), wind speed (WS), albedo (Alb), and land surface temperature (LST) surrounding Meyghan wetland, Iran. The dashed vertical line in each panel indicates the statistically determined break point. Mean ± SD (Standard Deviation) for the total period, the pre-change, and the post-change point period are shown with black, blue and red colors, respectively.**

**
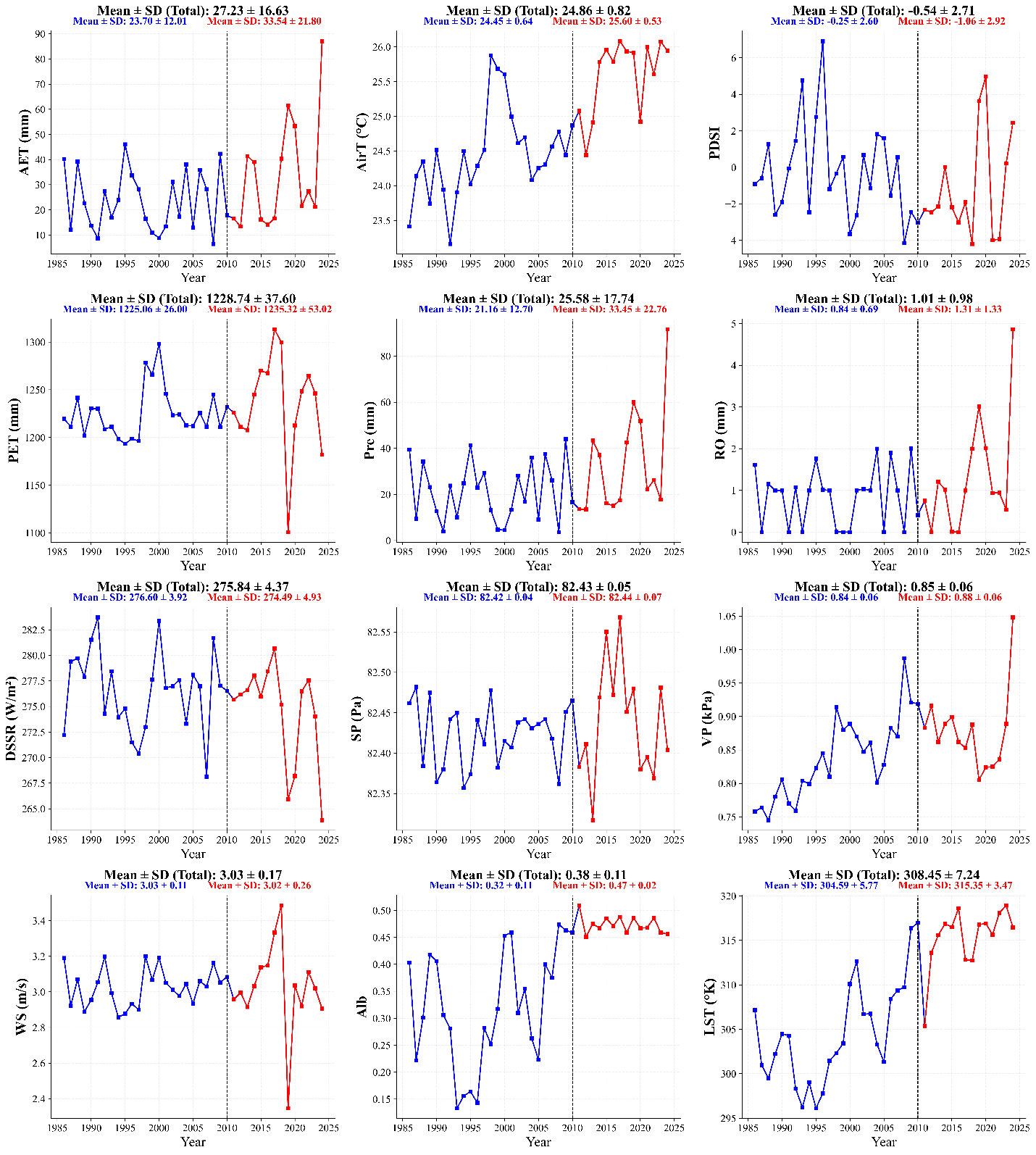
**

**Fig.S7. Seasonal changes during dusty seasons in AET (actual evapotranspiration), AirT (air temperature), Palmer drought severity index (PDSI), potential evapotranspiration (PET), precipitation (Pre), runoff (RO), Downward surface shortwave radiation (DSSR), surface pressure (SP), vapor pressure (VP), wind speed (WS), albedo (Alb), and land surface temperature (LST) surrounding Bakhtegan-Tashk wetland, Iran. The dashed vertical line in each panel indicates the statistically determined break point. Mean ± SD (Standard Deviation) for the total period, the pre-change, and the post-change point period are shown with black, blue and red colors, respectively.**

**
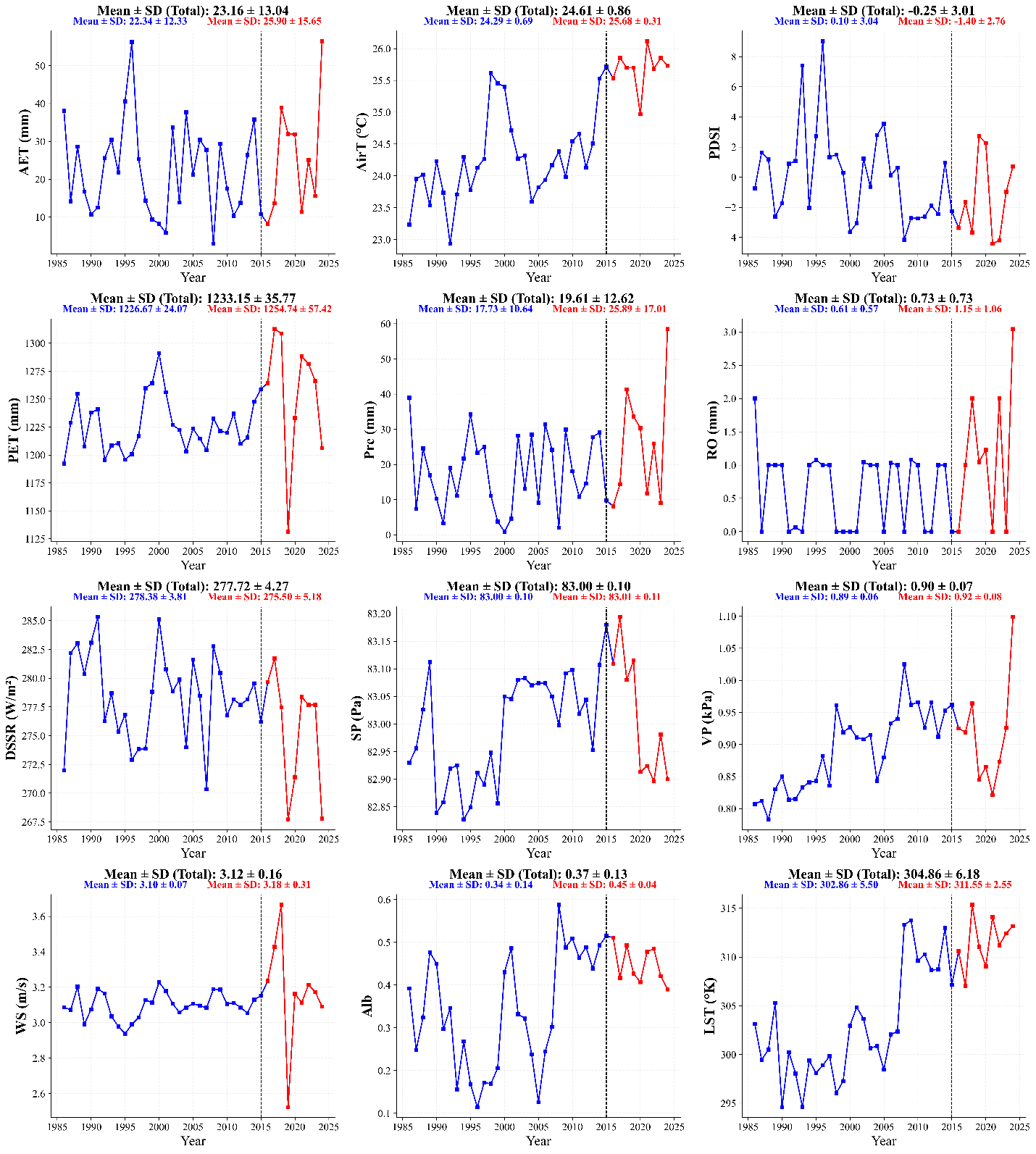
**

**Fig.S8. Seasonal changes during dusty seasons in AET (actual evapotranspiration), AirT (air temperature), Palmer drought severity index (PDSI), potential evapotranspiration (PET), precipitation (Pre), runoff (RO), Downward surface shortwave radiation (DSSR), surface pressure (SP), vapor pressure (VP), wind speed (WS), albedo (Alb), and land surface temperature (LST) surrounding Maharlu wetland, Iran. The dashed vertical line in each panel indicates the statistically determined break point. Mean ± SD (Standard Deviation) for the total period, the pre-change, and the post-change point period are shown with black, blue and red colors, respectively.**


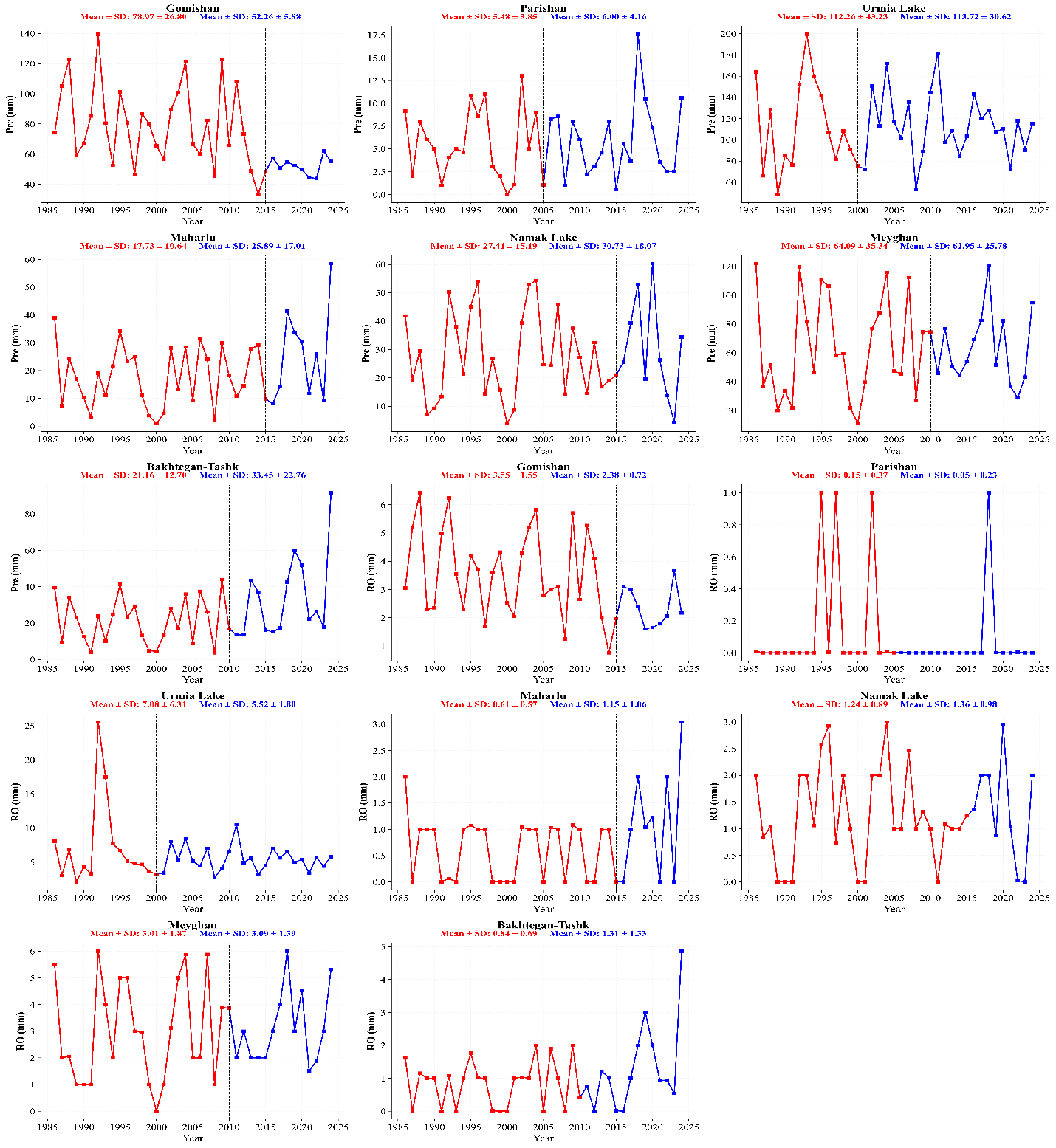


**Fig.S9. Seasonal variations in runoff (RO) and precipitation (Pre) during winter season for critical lakes and wetlands in Iran.**
